# The cellular triumvirate: fibroblasts entangled in the crosstalk between cancer cells and immune cells

**DOI:** 10.3389/fimmu.2023.1337333

**Published:** 2024-01-18

**Authors:** Adel Fergatova, Nesrine I. Affara

**Affiliations:** Carnegie Mellon University in Qatar, Doha, Qatar

**Keywords:** fibroblasts, immunosuppression, heterogeneity, reprogramming, cancer-associated fibroblasts

## Abstract

This review article will focus on subpopulations of fibroblasts that get reprogrammed by tumor cells into cancer-associated fibroblasts. Throughout this article, we will discuss the intricate interactions between fibroblasts, immune cells, and tumor cells. Unravelling complex intercellular crosstalk will pave the way for new insights into cellular mechanisms underlying the reprogramming of the local tumor immune microenvironment and propose novel immunotherapy strategies that might have potential in harnessing and modulating immune system responses.

## 1 Introduction

### 1.1 Origins of CAFs

Largely present in the tumor microenvironment (TME) are two types of fibroblasts: naïve fibroblasts and cancer-associated fibroblasts (CAFs). Naïve fibroblasts are essential in normal tissue homeostasis and function. They display different phenotypes and express numerous extracellular matrix (ECM) proteins depending on the host tissue type and state, which may explain the variations in ECM configurations (1). They normally become activated only in conditions requiring tissue remodelling, including wound healing and fibrosis, eventually being removed from the tissue by apoptosis to maintain homeostasis and the architecture of the environment (2). However, in the context of tumor progression, activated fibroblasts persist in the tissue and become one of the major contributors to tumorigenesis. They significantly promote cancer progression through numerous processes, including cell proliferation, angiogenesis, metastasis, in addition to infiltration and differentiation of other pro-inflammatory cells within the TME (3).

The origin of CAFs depends not only on the cancer type and progression but also on their localization within the TME. For example, an increasing number of studies suggest that breast CAFs originate from resident fibroblasts, bone marrow-derived mesenchymal stem cells, or cancer cells themselves (4), while in the context of pancreatic cancer, CAFs were derived from pancreatic stellate cells and mesenchymal stem cells (5). These findings highlight the uncertainty and the ambiguity associated with the origins of CAFs. For this reason, only the most reported cell types from which CAFs were derived in different cancer types will be further discussed in this article.

#### 1.1.1 Resident fibroblasts

Recent studies suggested that CAFs originate from resident fibroblasts in the vicinity of cancer cells, where they differentiate into activated fibroblasts via the orchestrated factors secreted by the tumor cells and other cells residing in the stroma (6). Growing evidence suggests that tumor-derived growth factors, such as transforming growth factor beta-1 (TGF-β1), platelet-derived growth factor (PDGF), and basic fibroblast growth factor (bFGF), convert resident fibroblasts into CAFs due to activation of autocrine activating signaling loops mediated by CAF-derived TGF-β and stromal cell-derived factor 1 (SDF-1) cytokines. Autocrine signaling enables a cell to produce a specific mediator, which upon binding and activating its own receptor, promotes expression of the same mediator, hence resulting in a repeated cycle of auto-stimulation (7). The abovementioned growth factors act together in auto stimulatory fashion and maintain the differentiation of resident fibroblasts into CAFs that would further acquire tumor-promoting phenotype. It has been reported that a significant amount of TGF-β released by cancer cells triggered TGF-β and SDF-1 production through TGF-β Receptor (TbR)-Smad signaling, where Smads, small intracellular effector proteins, activated by TGF-β regulate intracellular TGF-β signaling (8). In turn, activation of SDF-1 signaling has been shown to contribute to the sustained overexpression of TGF-β. These findings support the existence of direct interactions between tumor cells and activated fibroblasts, hence fostering differentiation and acquisition of an activated fibroblast phenotype (9).

#### 1.1.2 Bone marrow-derived mesenchymal stem cells

Another source of CAFs includes bone marrow-derived mesenchymal stem cells (MSCs), a subset of stem cells within the bone marrow stroma that can differentiate into cells of connective tissue or self-renew. Even if the recruitment of these cells into primary tumor sites and their contribution to CAF activation is not entirely known, growing evidence suggests that CXC motif chemokine ligand 16 (CXCL16) is responsible for MSC recruitment and their subsequent conversion into CAFs. To illustrate this novel recruitment and differentiation of CAFs, specific examples are included here: In the case of prostate cancer, CXC motif chemokine receptor 6 (CXCR6) signaling mediated by CXCL16 supports the migration of MSCs, which is abolished following the knockdown of CXCL16 (10). Other studies on prostate cancer suggest that chemoattractant factor such as TGF-β1 induces the migration of MSCs and drive their trans-differentiation into CAFs. It was documented that TGF-β1, whether tumor or stroma-derived, is essential for the differentiation of MSC into CAF-like cells. The TGF-β1 blockade impaired MSC mobilization and suppressed prostate carcinoma progression (11).

#### 1.1.3 Epithelial cells

Not surprisingly, CAFs can also arise from epithelial cells through EMT (epithelial-mesenchymal transition), where cells lose their epithelial characteristics and acquire mesenchymal-like characteristics, i.e., becoming more like fibroblasts. Mechanisms that mediate this transition are unclear; however, recent research points to matrix metalloproteinase-3 (MMP-3). Indeed, mouse mammary epithelial cells treated with MMP-3 exhibited downregulation of epithelial markers such as loss of E-cadherin, increased invasiveness, and overexpression of mesenchymal markers, including vimentin, smooth muscle actin (SMA), Snail, collagen A1, and fibronectin, suggesting their reprogramming into a more migratory phenotype. These drastic effects of MMP-3 may correlate with excess production of reactive oxygen species (ROS), causing DNA damage and genomic instability. It was suggested that MMP-3 promotes the expression of an alternatively spliced form of Rac1 in epithelial cells, which further results in the production of ROS. In this way, MMP-3-induced Rac activity contributes to the alteration of epithelial cells into a migratory phenotype similar to CAFs (12).

#### 1.1.4 Endothelial cells

A similar process has been documented in endothelial-to-mesenchymal transition, where resident endothelial cells change phenotypically from an organized cell layer to a mesenchymal phenotype characterized by loss of cell-cell junctions, endothelial markers, and acquisition of invasive phenotype. Interestingly, these endothelial cell-derived CAFs were recognized as a different population of cells that express both endothelial markers such as CD31 and mesenchymal markers such as vimentin, fibroblast specific protein 1 (FSP1) or alpha-smooth muscle actin (αSMA). On the other hand, recent studies reported that approximately 40% of studied FSP1-positive CAFs were also CD31 positive, which may imply that 40% of CAFs might be derived through endothelial-to-mesenchymal transition, hence acquiring fibroblast-like phenotype (13). Recent studies indicate that TGF-β1 mediates lung-derived endothelial cells’ transition into a mesenchymal phenotype (14). These studies suggest that endothelial-to-mesenchymal transition could be another unique mechanism for the fast accumulation of CAFs in the vicinity of the TME.

### 1.2 The challenging heterogenous nature of CAFs

**Figure 1:**
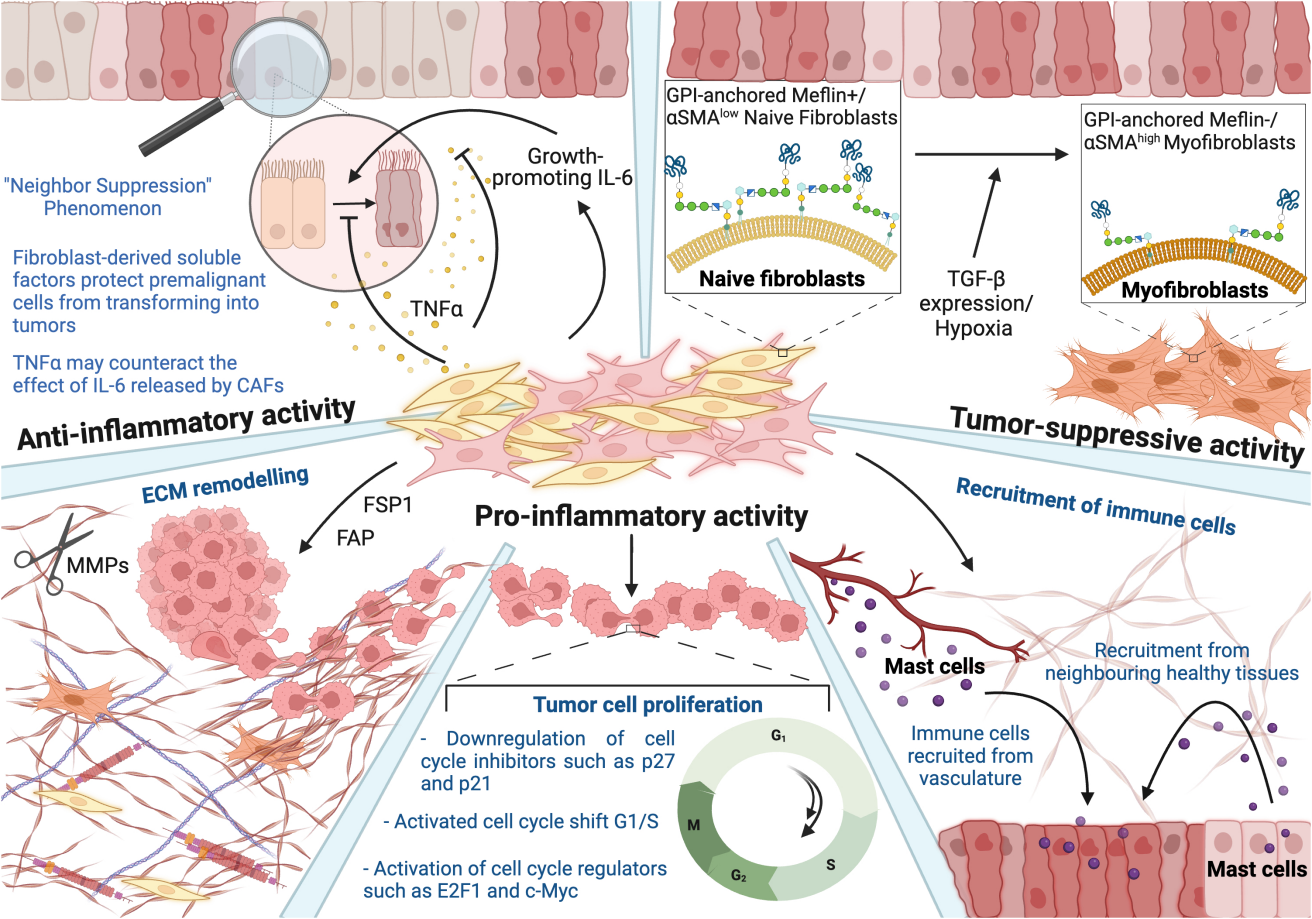
Heterogenous nature of fibroblasts reflected in their anti-inflammatory, tumor-suppressive, and pro-inflammatory activity. Anti-inflammatory: Naïve fibroblasts (yellow) inhibit the growth of adjacent abnormal or transformed cells, which is referred to as a “neighbor suppression” phenomenon. Fibroblast-derived soluble factors suppress the transformation of premalignant cells into tumors. TNFa may counteract the effect of CAF-derived IL-6 at the very early stages of carcinogenesis. Tumor-suppressive: The study showed that tumor-suppressive activity of Meflin, a glycosylphosphatidyl inositol-anchored protein, and low levels of αSMA in CAFs result in cancer-restraining activity. However, the expression of αSMA was also observed to be tumor-suppressive in the case of pancreatic ductal adenocarcinoma progression, stressing the duality in the functionality. Pro-inflammatory: CAFs contribute to various hallmarks of cancer, including its involvement in ECM remodeling, enhanced tumor cell proliferation, and recruitment of immune cells. This figure was designed by using graphic elements from BioRender.

CAFs are usually described as spindle-shaped cells that are negative for epithelial, endothelial, and leukocyte markers. It is important to note that many studies classify tumor and other cells differentiated into fibroblast-like or mesenchymal states undergoing epithelial-mesenchymal transition (EMT) or endothelial-mesenchymal transition (EndTM) as CAF population (15). In addition, fibroblasts are known to express a whole range of different biomarkers, including αSMA, vimentin, FSP1 and others. Using single-cell RNA sequencing and bioinformatics tools, scientists had hope in clustering CAFs into multiple subsets based on gene-expression patterns. In particular, a recent study reported the existence of at least four spatially and functionally distinct phenotypes within breast tumors, which were characterized based on differentially expressed genes that were defined in agreement with gene ontology (GO) classification. Population 1, which was termed vascular CAFs (vCAFs), was defined based on the expression of 150 genes that have been shown to be significantly enriched for vascular development and angiogenesis GO sets. An additional population was defined by the unique expression of 250 genes related to ECM and EMT and was depicted as matrix CAFs (mCAFs). Another set of cell-cycle related genes dominated in CAF Population 3, which identified the majority of cells in the G2, M, or S phase of the cell cycle, hence were termed as cycling CAFs (cCAFs). However, this CAF population was later found to represent a subpopulation of vCAFs that were actively proliferating (16). Interestingly, vCAFs shared many marker genes with pericytes, including platelet-derived growth factor receptor beta (PDGFRβ), Chondroitin Sulfate Proteoglycan 4 (CSPG4), and regulator of G protein signaling 5 (RGS5), which further supported its association with vascularization and tumor invasion. On the other hand, mCAFs expressed a wide range of matrix components, displaying a high association with a stroma-derived invasion like signature (17). Lastly, Population 4 was labeled as developmental CAFs (dCAFs). This population was distinguished by the expression of genes associated with stem cells, including Sry-type HMG box 9 (Sox9) and Sox10, among others. Notably, dCAFs are thought to originate from tumor cells that have undergone EMT, suggesting that during later stages of tumor progression, tumor cells may dedifferentiate into stem-like cells, conferring them with the ability to transdifferentiate into dCAFs. Collectively, the existence of subclasses of breast CAFs that may have derived from distinct cellular origins hints at a dynamic nature of the CAF phenotype rather than a fixed and one well-defined cell type population. Indeed, attempts to characterize this unique population based on its transcriptional patterns and abundance were disappointing since those markers are not specific to certain subpopulations and have not confirmed definitive functional validation. Assuming that function might be solely dictated by transcript abundance may lead to invalid presumptions and does not take into consideration post-transcriptional control or cell-to-cell interaction within the TME (15). Despite the recent optimism that CAFs represent a viable and novel therapeutic target, the literature has been replete with clinical trial failures or even acceleration of cancer progression due to an inadequate understanding of the functions of CAF subpopulations in cancer progression and the failure inaccurately characterizing these subpopulations. Moreover, their dynamic and interchangeable shifts between several phenotypes might be a possible explanation for their highly plastic and heterogenous nature (15). Taking this into consideration and instead of counting solely on expression patterns of certain markers, we opted to categorize the CAF subpopulations into three subgroups based on their functionality (**Figure 1**) –anti-inflammatory, pro-inflammatory, and tumor-suppressive – which may further explain and imply their recruitment/origin, as well as differential transcriptome.

#### 1.2.1 Anti-inflammatory activity

Naïve fibroblasts are essential in normal tissue homeostasis and function. They display different phenotypes and express numerous ECM proteins depending on the host tissue type and state, which explains variations in ECM configurations according to the tissue localization and type. Interestingly, the anti-inflammatory activity of normal fibroblasts is considered another prominent mechanism against cancer development and progression, specifically inhibition of the growth of adjacent abnormal or transformed cells. Scientists discovered that fibroblasts inhibit their growth upon contact with cancer cells, hence labelling this type of interaction as a “neighbor suppression” phenomenon (18). It was observed that fibroblast-derived soluble factors such as TGF-β, tumor necrosis factor alpha (TNFα), and interleukin-6 (IL-6) coordinate contact-independent suppression of tumor growth in a paracrine manner, thus suggesting that the default function of normal fibroblasts is to alleviate the transformation of normal or pre-malignant cells into tumor ones.

Depending on its concentration, TNFα may counteract the growth-promoting effect of IL-6 released by CAFs, at the very early stages of carcinogenesis. It is worth mentioning here that TNFα has already been tested in clinical trials and proved successful when combined with γ-irradiation. Hence, the study suggests that TNFα-derived fibroblasts may represent a novel drug design for cancer therapy (19).

Similar outcomes were observed in a study where tumor cells were co-cultured with freshly isolated mouse fibroblasts. Scientists noticed that following separation, tumor cells immediately regained their abnormal growth, suggesting the dominance of an anti-inflammatory effect of fibroblasts on tumor cells when in physical proximity to each other. These results further imply that these effects are transient and are reversed upon the removal of the NAFs (20). Subsequent studies further deciphered the underlying mechanisms and reported a progressive elongation of the cell cycle, which culminated in arrest in G2/M in the transformed cells (20). Collectively, these findings suggest that close cell contact is an important factor in controlling tumor cell growth; however, limiting this inhibition to special regions within the TME where NAF-tumor cells are in close proximity. Alternatively, fibroblasts have been shown to acquire their full inhibitory capacity only when in close contact with the tumor cells within the TME due to the secretion of soluble factors that are inducible only following the co-culturing of both cell lines. Thus, it was further suggested that NAF-mediated inhibition is facilitated by the secretion of NAF-derived soluble factors resulting from the tumor cell-NAF cross-talk (21).

Considering the predominant inhibitory ability of fibroblasts, it may be worth exploring in the future sorting the fibroblasts from different cell types, cancer stages and delineating their inhibitory potential as weak versus a strong one. So far, there has been only one study that used a similar approach, where five hundred heterotypic cell combinations and fibroblasts originating from different sites such as skin prostate were evaluated. For instance, these comparisons revealed that skin-derived fibroblasts are stronger inhibitors than other organ-specific fibroblasts. Interestingly, a negative correlation was observed between fibroblast-mediated inhibitory activity and age of patients, where fibroblasts originating from adult patients were reported to be less inhibitory (22).

#### 1.2.2 Tumor-suppressive activity

Recent studies have highlighted the ability of CAFs to also possess the capability of restraining carcinogenesis. The suppressive nature of CAFs was explored in pancreatic ductal adenocarcinoma (PDAC), specifically the Sonic Hedgehog (SHH) signaling pathway that had gained scientists’ attention. SHH contributes to the differentiation motility of human pancreatic stellate cells and the formation of desmoplasia, which normally mediates pro-tumorigenic effects and creates a barrier to the delivery of therapeutic compounds. Remarkably, it was observed that genetic depletion of αSMA positive-CAFs and blockage of the SHH pathway resulted in the progression of PDAC, suggesting a possible suppressive effect of CAF. These results led to the current view of CAFs as both tumor-promoting and tumor-restraining subpopulations of cells. Given these observations, αSMA represents a candidate marker of cancer-restraining CAFs. However, αSMA expression has been reported in a wide variety of cells, including tumor-promoting CAFs, again complicating the idea of fibroblast heterogeneity and hence delineating their functional significance (23).

Other studies focused on the expression of Meflin in CAFs, a glycosylphosphatidyl inositol-anchored protein that is a specific marker of mesenchymal stromal/stem cells (**Figure 1**). It was found that Meflin inhibits PDAC progression, presumably by decreasing the expression of αSMA in CAFs and changing the collagen configuration in the stroma. Meflin positive-CAFs express low levels of αSMA, yielding αSMA^+/low^ Meflin^+^ CAFs phenotype, inducing cancer-restraining action. Due to its tumor-suppressive activity, Meflin is usually downregulated with patient’s age, and hypoxia, TGF-β signaling and promoting differentiation of pancreatic stellate cells into αSMA-positive myofibroblasts. This may suggest that Meflin can suppress myofibroblastic differentiation and mediate ECM remodelling, hence correlating with favorable outcomes in patients with PDAC. Given the tumor-suppressive activity of Meflin, scientists are now interested in the possibility of reversing the phenotype of cancer-promoting CAFs by restoring Meflin expression (23).

CAFs were also found to promote metastatic growth in the liver while simultaneously restricting it, supporting the notion of the dual nature of CAFs. Previous studies suggest that a dominant biological effect of CAFs on ECM is the production of type I collagen, which increases stiffness and mechanosensitive signaling. Indeed, it has been shown that the mechanical restriction of tumor cells by collagen type I overrides tumor-promoting activity of CAFs. However, the tumor-promoting effect still dominates the tumor-suppressive ones due to other CAF-secreted factors (24).

#### 1.2.3 Pro-inflammatory activity

**Figure 2:**
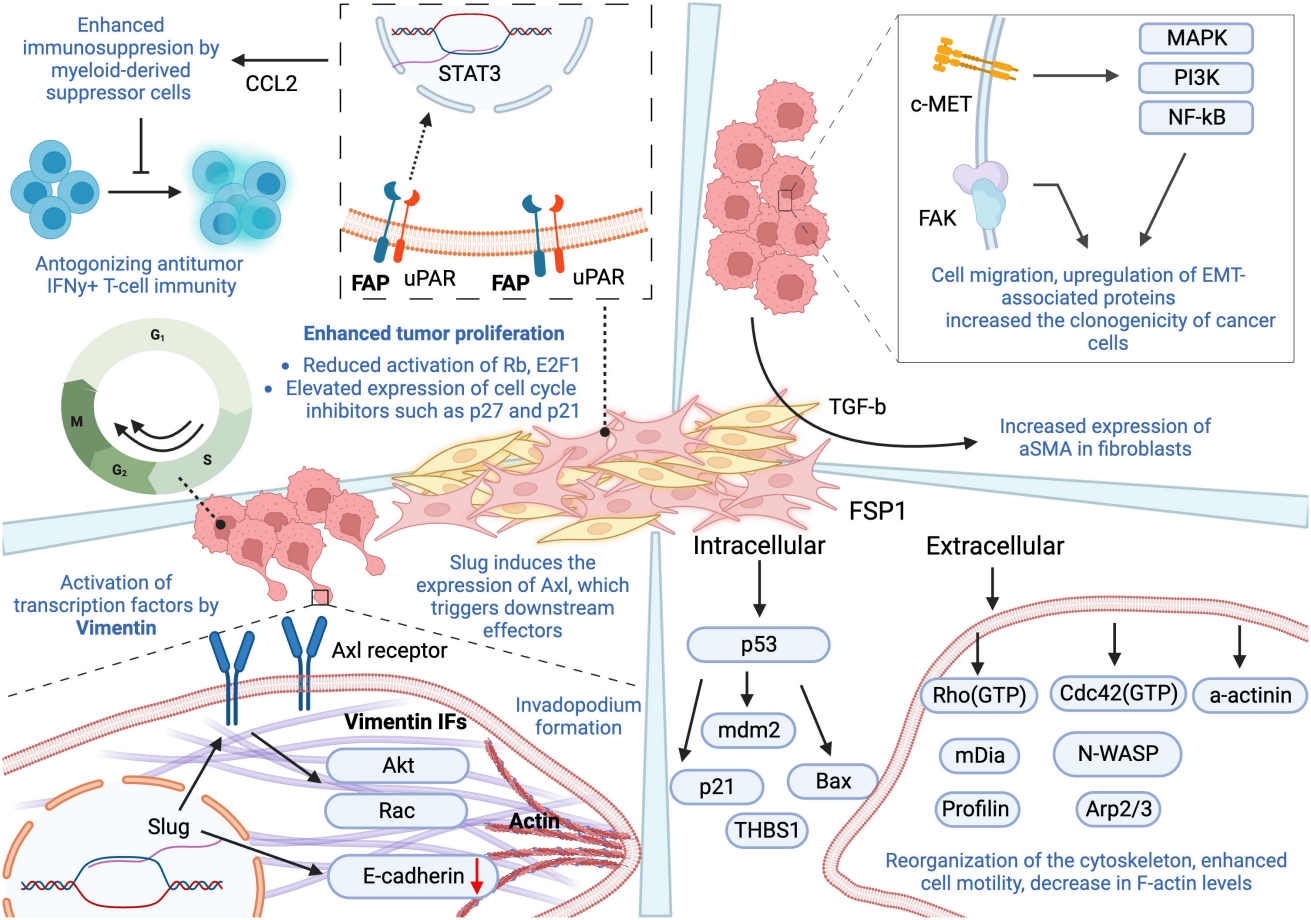
Pro-inflammatory activity of four fibroblast-derived markers and their contribution to hallmarks of cancer. Fibroblast Activation Protein-a: Despite short intracellular domain of FAP, its close association with uPAR results in activation of downstream effectors, leading to the activation of transcription factor STAT3. FAP-positive CAFs are a major source of CCL2 that enhances immunosuppressive activity mediated by myeloid-derived suppressor cells, in addition to overexpression of genes responsible for antagonizing anti-tumor IFNγ+T-cell immunity. FAP expression also contributes to tumor cell proliferation by reducing activation of Rb and E2F1, and increased expression of cell cycle inhibitors, including p27 and p21. Vimentin: It has been shown to be essential for Slug-induced migration and increased expression of Axl. Slug downregulates E-cadherins, while activated Axl triggers other downstream proteins, such as Rac and Akt, contributing to cell motility and EMT. The assembly of vimentin is vital for the formation of invadopodium. Alpha Smooth Muscle Actin (αSMA): The expression of αSMA in fibroblasts was observed following the treatment with TGF-β. In addition, it plays a role in inducing EMT and increased migration. αSMA upregulates the transcription of EMT-associated proteins, such as c-MET and FAK, which further trigger numerous pathways that induce proliferation, cell motility, cell survival, and EMT. Fibroblast-Secreted Protein 1(FSP1): FSP1 is associated with stabilization of p53 and modulation of genes regulated by it, including mdm2, Bax, p21, and thrombospondin-1 (THBS1). It also stimulates cell motility through reorganizing actin cytoskeleton and moderating the activity of its regulators. Extracellular FSP1 decreases the levels of F actin and various cytoskeleton organization regulators, evident from a decrease level of RhoA, mDia, and profilin. Its involvement with Cdc42, N-WASP, and Arp2/3 results in the initiation of new filament growth. This figure was designed by using graphic elements from BioRender.

The role of CAFs in cancer has been mostly associated with a tumor-promoting phenotype. The primary functions of activated fibroblasts are hijacked and enhanced, fostering an immunosuppressive and pro-inflammatory microenvironment. CAFs were observed to contribute to almost all hallmarks of cancer, thus supporting tumor proliferation, invasion, and metastasis (25). Considering the heterogeneity of this population mentioned above, markers are expressed in different levels and ratios depending on cancer type, stage, and progression. For this reason, this review will focus on the most frequently discussed four markers (**Figure 2**), delving into cell signaling and their contribution to hallmarks of cancer, which depict the pro-inflammatory activities of CAFs in a more comprehensive and insightful manner.

##### 1.2.3.1 Marker 1: fibroblast activation protein-α

FAP is a serine protease that cleaves peptide bonds between certain amino acids, modifying various bioactive molecules and possessing unique endopeptidase activity that enables it to cleave collagen type I and gelatin (26). It is not normally expressed in healthy adult tissue, with the exception of wound repair processes. However, recent studies revealed that in the disease state, FAP is localized to the surface of fibroblasts in the stroma, which accounts for more than 90% of tumor mass in various cancers, including malignant breast, colorectal cancer, skin cancer and bone sarcomas. Overall, overexpression of FAP has been associated with poor prognosis in multiple types of cancer (27).

By activating signal transducer and activator of transcription 3 (STAT3), which is an important transcription factor in tumor-associated inflammation, FAP has been shown to mediate the inflammatory effects of CAFs. Although FAP with a short intracellular domain is unlikely to initiate activation of the signaling pathway, however, its close association with urokinase plasminogen activator surface receptor (uPAR) is crucial for it to mediate gene expression of important inflammatory genes. More specifically, uPAR causes the initiation of activation of other downstream proteins that are known to participate in tissue remodelling and cancer development, leading to the activation of transcription factor STAT3. As a result, FAP-positive CAF acts as a major cell source of C-C motif chemokine ligand 2 (CCL2), which enhances immunosuppression through tumor- and inflammation-promoting myeloid-derived suppressor cells (MDSCs) (28). Along the expression of FAP, macrophage frequency and expression of genes related to MDSCs have been shown to increase significantly, leading to tumor infiltration of MDSCs and T cells. Furthermore, FAP-positive CAFs upregulate the expression of immunosuppressive genes of MDSCs, making their ability to inhibit T-cell proliferation more effective and antagonizing anti-tumor interferon-gamma (IFNγ)+T-cell immunity (28).

FAP also functions as an active serine protease that is capable of degrading type I collagen, hence contributing to alterations in ECM structure and composition, thus enhancing the ECM permissiveness and invasiveness. Here, we will discuss these effects in the context of pancreatic cancer progression. Interestingly, FAP+ fibroblasts produced ECM fibers oriented in parallel patterns, which are reminiscent of previously reported patterns in human pancreatic adenocarcinoma cells. This observation supports the possibility of FAP+ fibroblast-derived matrices, therefore efficiently recapitulating tumor ECM and perhaps producing new matrices that may facilitate tumorigenesis. Moreover, the expressions of proteins important for tumorigeneses such as αSMA, fibronectin, and collagen I were significantly upregulated in the FAP+ matrix. In this way, FAP fosters changes in ECM architecture, culminating in increased ECM permissiveness and facilitating tumor invasiveness (29).

Moreover, fibroblastic expression of FAP has been shown to interfere with cell cycle progression, leading to an increase in tumor cell proliferation. Indeed, co-culturing tumor cells with FAP-expressing fibroblasts significantly increased the fraction of tumor cells in the S/G2/M phases. On the other hand, FAP knockdown blocked the transition of tumor cells from G1 to S phase. This was paralleled with reduced activation of retinoblastoma (Rb), E2F transcription factor 1 (E2F1), and elevated expression of cell cycle inhibitors such as p27 and p21. This suggests that FAP+ fibroblasts mediate phosphorylation of Rb, which in its unphosphorylated form arrests cells in the G1 phase, thus supporting tumor cell proliferation (30).

##### 1.2.3.2 Marker 2: vimentin

Although vimentin, a major member of the intermediate filament proteins, was first described with a limited number of roles in physiological and pathophysiological contexts, many recent studies highlight its normal expression in a wide range of cells with a mesenchymal origin, including macrophages, neutrophils, and fibroblasts (31). However, changes in expression patterns of intermediate filaments are associated with tumor progression and growth, particularly leading to increased cellular migration and invasion (32).

CAF invasion has been shown to be mediated by overexpression of vimentin (33). Using 3D culture models, co-culturing CAFs with down-regulated vimentin along with lung adenocarcinoma cells results in spheroids with reduced formation of invasive branches, suggesting an attenuated ability of tumor cells to migrate out of the spheroids. In this way, fibroblastic overexpression of vimentin regulates CAF invasion, and cancer cell-CAF interaction results in decreased spheroid circularity (33). Almost all studies that have evaluated the role of vimentin during tumorigenesis used epithelial cells. Similar studies evaluating the role of vimentin in fibroblast subpopulations have yet to be established to discern whether these functions are cell or cancer type specific.

The co-localization of vimentin and microtubules suggests its role as a linker between actin and the microtubules, maintaining cytoskeleton structure and mechanical force stability, contributing to cell polarity and motility (34). In addition, vimentin has been shown to be necessary for Slug-induced migration and increased expression of Axl, which contribute to increased cell motility and EMT. Indeed, vimentin regulates the expression of the EMT-related transcription factors including Slug, which results in markedly elongated morphology of tumor cells and induces the expression of Axl, a receptor tyrosine kinase that is stimulated by the secreted protein growth arrest-specific 6 (Gas6). The E-cadherin repressor, Slug, downregulates it. This activated signaling pathway triggers other important downstream proteins, such as Rac and Akt, which are regulators of cell motility (35).

The assembly of vimentin into the filament is also critical for the formation of invadopodia, specialized dynamic actin-rich membrane protrusions possessing degradative activity that allow cells to overcome a dense scaffold within the microenvironment (36). Vimentin forms a network from the perinuclear region towards the cell periphery, becoming an anchor for invadopodia that downwardly protrudes from it. Plectin, an intermediate filament-associated multidomain protein and member of cytolinker proteins, anchors invadopodia to vimentin by crosslinking actin filaments of invadopodia with vimentin (37).

##### 1.2.3.3 Marker 3: fibroblast-secreted protein

Fibroblast-secreted protein (FSP1, also called S100A4), a calcium-binding protein, is highly specific for fibroblasts and is associated with the conversion of epithelial cells to a fibroblast phenotype. It is known to form homodimers and interact with different proteins in a calcium-dependent way. FSP1 is normally involved in the regulation of cell growth, cell-cell communication, contraction, and cell motility (38). Having no known enzymatic activity, localization of FSP1 (intra- or extracellular) is undoubtedly critical. In the extracellular space, FSP1 interacts with cell surface receptors and initiates signaling pathways. Intracellularly, it exists predominantly as a symmetric homodimer, facilitating the binding with target proteins. The intracellular translocation of the protein has also been reported to occur under the extracellular presence of the same FSP1 protein. Similar to vimentin, most of these studies have been done using tumor cells rather than fibroblasts. For instance, nuclear expression of FSP1 correlates with poor prognosis in ovarian carcinomas, which suggests the potential importance of localization of FSP1.On the other hand, treatment of tumor cells with the FSP1 resulted in the stabilization of p53 and modulation of genes regulated by it, including mouse double minute 2 (mdm2), Bcl-2-associated x (Bax), p21, and thrombospondin-1 (THBS1). It is not known whether these proteins interact in the cytoplasm and/or in the nucleus (39).

It has been also suggested that FSP1 protein might play a role in tumor progression by increasing the motility of endothelial cells, hence modulating angiogenesis. Moreover, it is well known that the MMP expression level correlates with metastatic potential in advanced cancer. In response to FSP1 treatment, transcription of MMP-11, MMP-13, and MMP-14 was increased, in addition to the activation of the transcription factor, nuclear factor kappa B (NF-kB). There is a possibility that the stimulation of motility and invasion by FSP1 is interposed by modulation of MMP expression, which in turn mediates the degradation of ECM (40).

Another way of stimulating cell motility is modifications at the levels of organization of the actin cytoskeleton and its different regulators. For instance, proteins rhodopsin (Rho), Rac and cell division control 42 (Cdc42) are involved in the regulation of actin polymerization dynamics, which take part in the formation of different structures such as lamellipodial protrusions or filopodium formation. Interestingly, the expression of extracellular FSP1 decreases the levels of F-actin and various operators responsible for the regulation/organization of actin, which was evident from a decreased expression of RhoA, mDia and profilin responsible for the polymerization of actin into stress fibers. Moreover, upon FSP1 treatment, a decrease in a-actinin was observed, which plays the role of a linker between cytoplasmic integrin subunits and filamentous actin. Its decrease is associated with enhanced cell motility. In addition, the involvement of Cdc42 and N-WASP was shown after exposure to FSP1. These proteins and the actin related protein 2/3 (Arp2/3) complex result in the initiation of a new filament growth (41).

##### 1.2.3.4 Marker 4: -smooth muscle actin

α-Smooth muscle actin (αSMA, ACTA2) is the actin isoform that plays an important role in tissue fibrogenesis. In addition to the fibroblasts, it is normally expressed in smooth muscle cells and blood vessels. Myofibroblasts are interesting given that they are not part of normal tissue and appear only following certain tissue injuries. Interestingly, they are also associated with inflammation, fibrosis, and carcinogenesis. Sometimes, the literature would refer to CAFs as activated myofibroblasts due to their high expression levels of αSMA (8). It was observed that the expression of αSMA was increased in fibroblasts following the TGF-β treatment. TGF-β is an inflammatory cytokine released by tumor cells that are involved in various stages of tumorigenesis, including its role in modulating growth of fibroblasts, suppression of lymphocyte proliferation, and cell survival. When TGF-β is bound by a dimeric pro-protein called the latency-associated peptide (LAP), it is considered in its latent form. The additional binding of the latent TGF-β binding protein (LTBP) makes up the large latent complex (LLC), which is often bound to extracellular matrix components like collagen. Activation of TGF-β involves the release of the LLC from the matrix and following release of TGF-β through a conformational change or proteolysis of the LAP (42). Activated TGF-β binds to TGF-receptor on the surface of fibroblasts and activates a signaling pathway that increases the expression of αSMA. These results suggest that overexpression of αSMA is a marker of activation of fibroblasts into myofibroblast phenotype. TGF-β expression may initiate the activation of the process through αSMA expression (8).

Although studies evaluating αSMA in fibroblasts are limited, we can draw parallels with the role of αSMA in the context of tumor cells. Indeed, αSMA mediates tumor cells metastatic potential, migration, and clonogenicity. Clonogenicity is one of the phenotypes of stemness, which is critical for establishing distant metastasis. It was observed that inhibition of αSMA downregulated the transcription of EMT-associated proteins such as c-MET and focal adhesion kinase (FAK), which usually mediate the increased migration and invasion properties of tumor cells. c-MET signaling activates numerous downstream pathways in epithelial cancer cells, such as mitogen-activated protein kinase (MAPK), phosphoinositide 3-kinase (PI3K), and NF-kB. FAK was also found to play an important role in TGF-β1-induced EMT. In this way, αSMA contributes to cell migration by upregulating EMT-associated proteins and increasing cancer cells’ clonogenicity. The study also highlights the possibility of the contribution of CAF-derived αSMA to similar processes, emphasizing its importance in the induction of EMT in different cell types (43).

### 1.3 Cross-talk between CAFs and immune cells

Very little is known about how immune cells are reprogrammed and how their interactions CAFs result in a microenvironment that is permissive for cancer progression. Hence, this article is centered around intercellular crosstalk between CAFs, different types of immune cells and tumor cells, delving deeper into cell signaling pathways and mechanisms underlying immunosuppression and evasion.

#### 1.3.1 Mast cells

**Figure 3:**
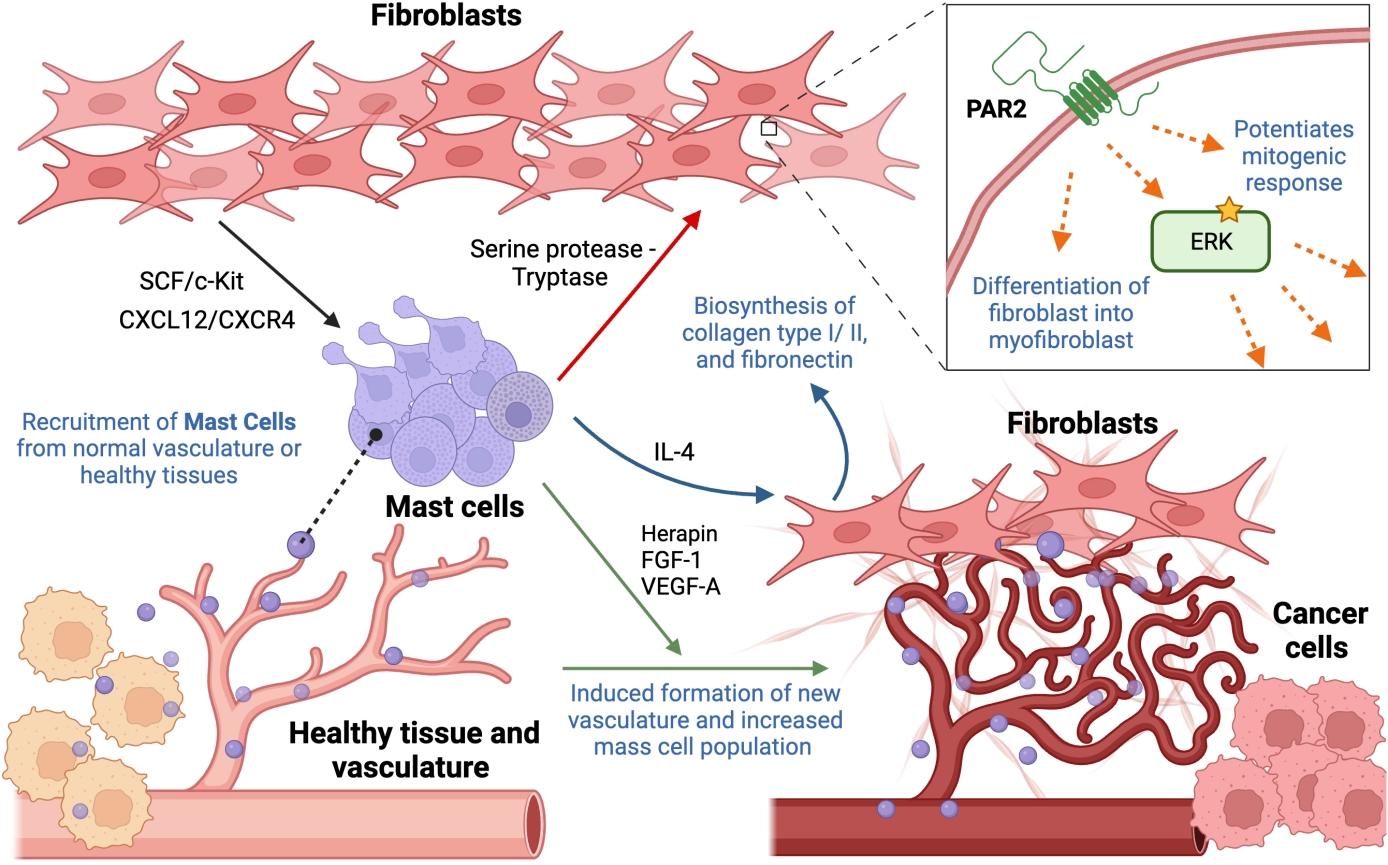
Cellular interactions between mast cells and fibroblasts contributing to hallmarks of cancer. Mast cells are recruited from neighboring tissues or vasculature to the tumor periphery by fibroblast-derived stem cell factor and CXCL12/CXCR4 interaction. Once migrated to the tumor site, there are multiple ways they interact with fibroblasts. Their crosstalk, involving serine protease (tryptase), increases ECM production, cell proliferation, fibroblast infiltration, in addition to triggering their differentiation into myofibroblasts, through activation of PAR2 and its downstream effectors. Mast cell-derived IL-4 results in enhanced proliferation of neighboring fibroblasts, stimulating biosynthesis of collagen type I and type III, and fibronectin. In addition, mast cells contribute to angiogenesis by secreting herapin, FGF-1, and VEGF-A, regulating integrity of existing and formation of new vessels. This figure was designed by using graphic elements from BioRender.

Mast cells originate from hematopoietic stem cells in the bone marrow. Mast cells can migrate into other tissues and differentiate into mature mast cells under the influence of different factors and cytokines, including IL-5, stem cell factor (SCF), and others (44). The cytoplasm of mast cell contains 50-200 large granules that store numerous inflammatory mediators, including histamine, heparin, proteases, and cytokines. They are usually located near blood vessels, lymphatics, and mucosal surfaces. Interestingly, mast cells are usually referred to as “orchestrators of anti-tumor immunity” because they directly impact tumor cells as well as immune and non-immune components, leading to a wide range of cancer-promoting or suppressive properties (**Figure 3**) (45).

Rather than infiltrating the tumor itself, mast cells are mainly localized to the tumor periphery. This specific localization suggests that their recruitment occurs either from resident mast cells migrating from neighboring healthy tissues or the recruitment of progenitor cells via normal vasculature to the tumor sites (46). In one study, bone marrow mast cells were cultured along with lung-derived fibroblasts, demonstrating that this particular interaction resulted in degranulation of mast cells. Eotaxin, a chemokine responsible for eosinophil recruitment, was secreted upon the interaction of the two cell populations. Mast cells and fibroblasts can generate substantial amounts of eotaxin, but the synergistic production of the chemokine through the intercellular interaction may suggest the importance of the presence of both. Moreover, the involvement of fibroblast-derived transmembrane SCF was observed to be necessary for their interaction and the maintenance of mast cells since it is responsible for binding mast cells to other cells and matrix. Interestingly, fibroblasts can produce both soluble and transmembrane SCF, where one drives eotaxin synthesis while the other contributes to persistent mast cell activation, respectively. The production of eotaxin as the result of their interaction suggests a novel relationship (47). Other studies also support these observations, reporting SCF as a mast cell growth factor and chemoattractant that stimulates directional mast cell activity. Moreover, it was reported that the SCF/c-Kit RTK signal is mainly responsible for the migration of mast cells into the tumor site as well as the functional activation and secretion of mediators. Interestingly, SCF acts on mast cells in a dose-dependent manner, where low levels induce migration and production of active MMP-9, and higher concentrations activate mast cells, resulting in the secretion of mediators. Hence, this feedback circle between mast cells and fibroblast-derived SCF may result in the recruitment and activation of mast cells in the TME (48). Moreover, it was suggested that CXCL12/CXCR4 interaction is also responsible for the migration of mast cells and their accumulation. A significant reduction in migration of mast cells was observed following decreased expression levels of CXCL12 or blocking of its cognate receptor, CXCL4 receptor (49). Collectively, the presence of several fibroblast-mediated ways to recruit mast cells points to redundancy in cellular pathway, posing a possible challenge to targeting the infiltration of mast cells.

Studies provide evidence that mast cells may affect the proliferation of neighboring fibroblasts in tissues by secreting IL-4, a cytokine usually released by T cells and mast cells, directly through IL-4 receptor expressed on fibroblasts. This effect is induced only when mast cells form intimate heterotypic cell-cell contacts with fibroblasts, which may be any type of transient or permanent physical interaction. Interestingly, IL-4 could not be detected in the supernatant after cell-cell contact of both cells, which may suggest that it may be secreted in small amounts by mast cells and strictly to intercellular space between fibroblasts and mast cells. IL-4 may be degraded or attached to other components of mast cells if it does not bind to its receptors on fibroblasts. It was further observed that the addition of IL-4 alone without any presence of mast cells still triggered fibroblast proliferation, further highlighting the importance of IL-4 as a paracrine mitogenic signal secreted by mast cells. It is important to mention that a mitogenic ability of mast cells was only observed in the presence of FGF, fibroblast growth factor. However, fibroblasts reached their maximal proliferation due to mast cell-derived IL-4 even in the presence of low concentrations of FGF, emphasizing that mast cells can influence the fibroblast population and potentiate its growth (50). Moreover, it was reported that IL-4 mediates the synthesis of type I and type III collagen and fibronectin in human dermal and synovial fibroblasts. Expression of these ECM proteins correlated with increased levels of their cognate mRNA levels (51).

Recent studies also suggested a novel player that may mediate fibroblast migration: Protease-activated receptor 2 (PAR2), which is a G-protein coupled receptor activated by serine proteases, leading to activation of downstream effectors such as ERK1,2. Its overexpression has been reported in fibroblasts and myofibroblasts, influencing fibroblast migration and ECM production. The activation of this receptor occurs through mast cell derived-serine protease and tryptase. Other studies suggest that tryptase/protease activated protein 2 (PAR2) interaction may also influence fibroblast differentiation into a subpopulation of myofibroblasts, resulting in overexpression of αSMA (52). More recent studies suggest the role of tryptase as a potent mitogen for fibroblasts *in vitro*. It was observed that tryptase potentiates the mitogenic response of fibroblasts to bFGF, epidermal growth factor (EGF) and insulin. However, underlying mechanisms have yet to be determined. Ongoing research suggests that the concentrations of tryptase used in these experiments can be readily achieved in the microenvironment of the degranulating mast cells. Hence, tryptase may stimulate DNA synthesis and enhance fibroblast proliferation (53).

Mast cells promote cancer growth and progression not only by affecting fibroblasts but also via interacting with other cells and structures within the TME. Mast cells have been long shown to reside and accumulate in the vicinity of blood vessels and are frequently associated with the formation of new vasculature. Mast cell population increases strikingly in the highly vascularized areas of certain tumors. Using a mouse model of squamous epithelial carcinogenesis, it was observed that mast cells secrete numerous factors to promote an angiogenic phenotype and accumulate around capillaries suggesting their striking role in angiogenesis (54). A more recent study on decreased efficacy of vascular endothelial growth factor receptor (VEGF(R))-targeted anti-angiogenic therapies due to the presence of mast cells also supports the notion of their pro-angiogenic role. They observed that the therapy had an anti-angiogenic effect only in the beginning, being gradually abrogated over time due to the mast cell-mediated restimulation of angiogenesis through upregulation of granzyme B, which is responsible for the liberation of pro-angiogenic factors other than those associated with the VEGF pathway, including FGF-1 and granulocyte-macrophage colony-stimulating factor (GM-CSF). Additionally, it was suggested that mast cells also release herapin, which is required for stabilization of FGF-1 dimerization. Despite the fact that therapy targets the VEGFA-VEGFR2 axis, mast cells induce the secretion of alternative factors that bypass it, emphasizing the notion of redundancy (55).

#### 1.3.2 Natural killer cells

**Figure 4:**
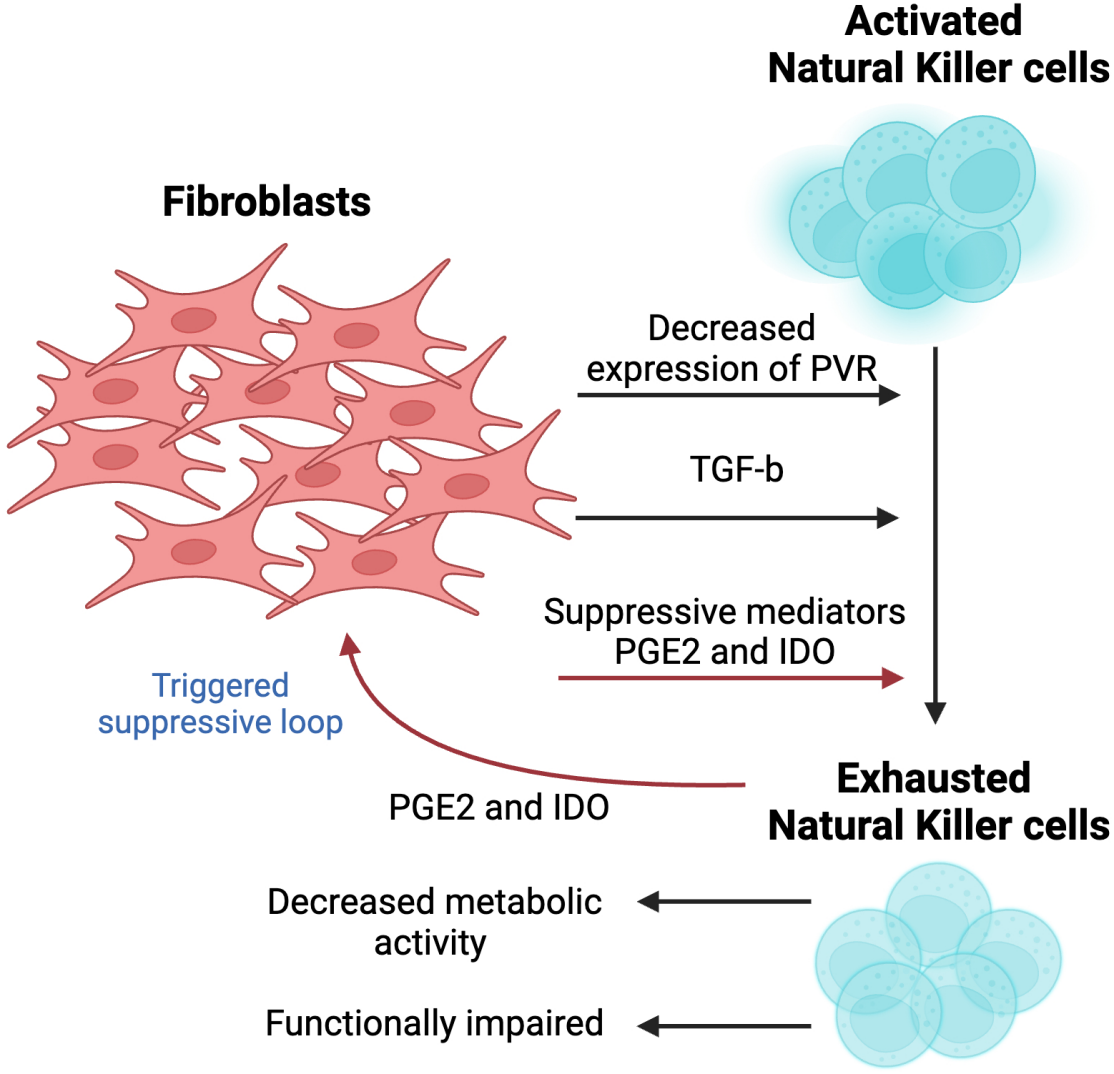
The effect of fibroblasts on the activity and cytotoxicity of natural killer (NK) cells. A crosstalk between two cell populations results in metabolically exhausted and functionally impaired NK cells. Fibroblast-derived suppressive mediators, including Prostagladin E2 (PGE2) and 2,3-dioxygenase-1 (IDO1), contribute to the exhausted phenotype. Since activity of NK cells is largely dependent on cognate ligands, fibroblasts decrease the levels of poliovirus receptor (PVR/CD155) to suppress NK cell-killing activity. *In vivo* studies showed that the activation of TGF-β signaling hampered the development of NK cells. This figure was designed by using graphic elements from BioRender.

In contrast to other immune cells, natural Killer (NK) cells require no MHC restriction or prior sensitization, hence shaping the early immune response. Indeed, NK cells do not require antigen specificity for activation, relying on the equilibrium between activating and inhibiting signals that are mediated by surface receptors, which interact with cognate ligands secreted by target cells, including virus-infected cells or tumor cells (56). NK cells secrete numerous inflammatory cytokines and chemokines, such as IFN-γ and TNFα, in a contact-dependent manner. Interestingly, NK cells recruited to a tumor site are dysfunctional, exhausted and metabolically impaired, enabling tumor cells to escape immune surveillance (57). The culprit cells mediating such suppression of NK immunity include CAF populations, which ultimately inhibit various pathways responsible for cell proliferation and functionality (**Figure 4**).

The activity of NK cells is influenced by the expression and stimulation of activating or inhibitory receptors on the cell surface, which makes it dependent on the expression of cognate ligands by target cells. Cell-to-cell interaction between NK cells and CAFs plays a crucial role in mediating CAF’s inhibitory activity, which is evident from the complete abolition of NK activity suppression when limited interaction was observed, further implying the importance of the physical close interactions between these two cell types (58). NK activity has been shown to be modulated by the expression of CAF-derived poliovirus receptor (PVR/CD155), the ligand of paired DNAM-1 (activating) and TIGIT (inhibitory) receptors. More specifically, reduced levels of PVR have been suggested as a mediator in CAFs-induced suppression of NK cells. Interestingly, naïve fibroblasts did not exert the same effect on NK cells, highlighting the unique role that CAFs may play in modulating the tumor immune microenvironment (58). While the mechanism underlying PVR downregulation remains unknown, it does not undermine its potential to be used as a potential approach to activate NK, providing a novel strategy to counteract tumor immunosuppression.

Additional mediators of CAF-triggered suppression of NK cells have been reported, including prostaglandin E2 (PGE2) and indoleamine 2,3-dioxygenase (IDO) (59). Interestingly, suppression of NK cells has been shown to be almost entirely abolished following the use of PGE2 and IDO inhibitors. This interaction between CAF and NK cells is bidirectional, where CAF-mediated deactivation of NK cells has been shown to trigger CAF cells to secrete high levels of PGE2 further, hence amplifying the magnitude of this suppressive loop (60). However, this paradoxical loop remains unclear and has yet to be further explored.

In addition, TGF-β hinders the activity of mammalian target of rapamycin (mTOR), a serine-threonine kinase that has been recently reported as a target of CAF-mediated suppression of the immune TME and widely known as a “molecular rheostat” of NK responsiveness (61). Some studies suggest that CAFs downregulate the expression of mTOR through TGF-β. Indeed, *in vitro* treatment of NK cells with TGF-β resulted in reduced activation of mTOR by blocking its IL-15-induced activation, resulting in decreased metabolic activity and growth of NK cells. A similar observation was made during *in vivo* studies, where activation of TGF-β signaling hampered the development of NK cells. An opposite effect characterized by enhanced cytotoxic activity of NK cells upon exposure to IL-15 was observed in response to TGF-β knockout. Moreover, its suppression in NK cells showed an improved ability of NK cells to restrict metastases in two tumor models in mice (62).

#### 1.3.3 Regulatory T cells

**Figure 5:**
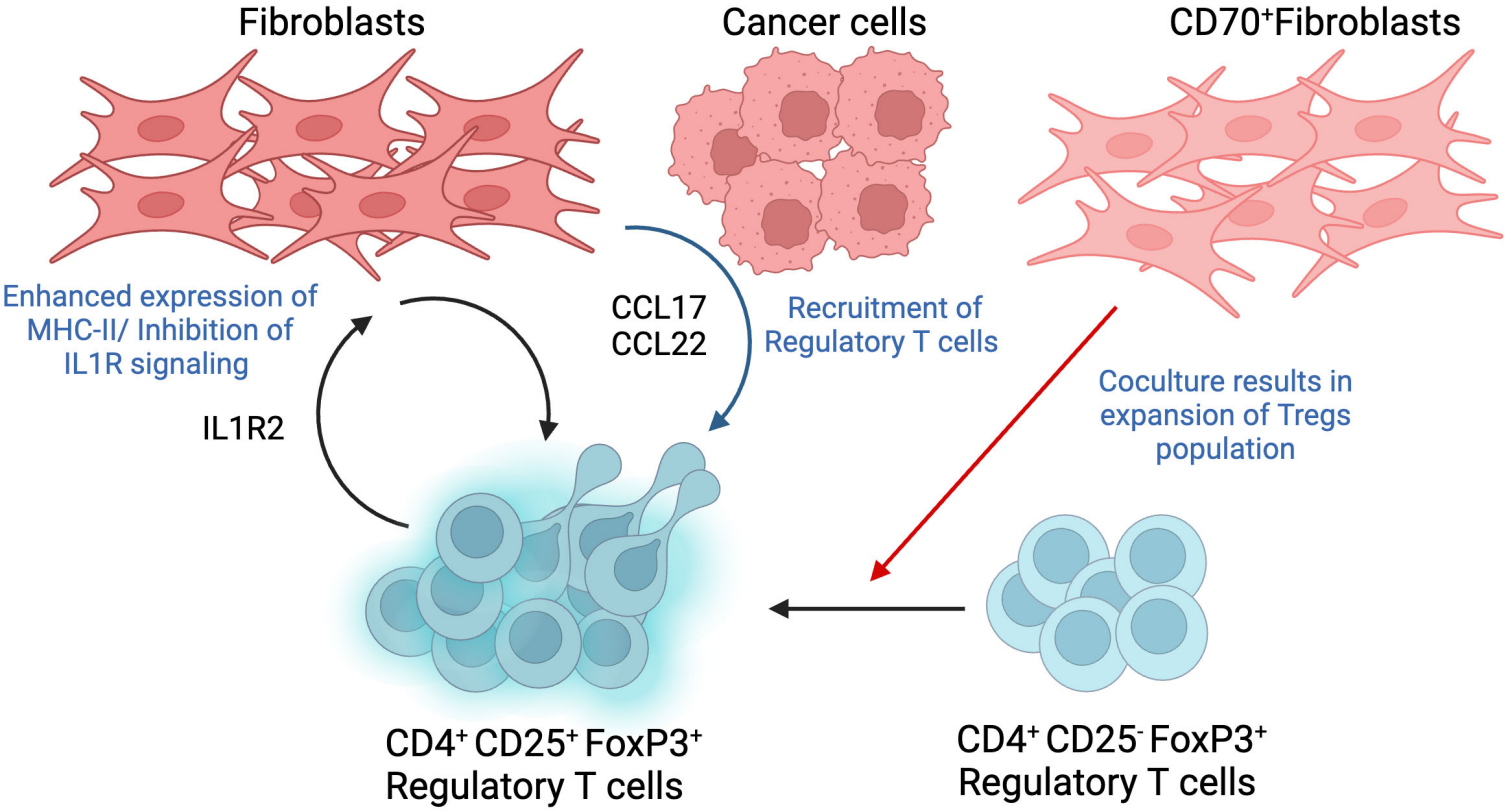
Interactions between Regulatory T cell (Tregs) and Fibroblasts. Tregs are identified as a CD4+CD25+FoxP3+ T cell population, where the expression of high FoxP3 shows a significant correlation with poor prognosis in different cancer types. Co-culture of CD70-positive CAFs with Tregs showed double fold increase in the cell population and promoted a shift from CD25-negative to CD25-positive phenotype. Fibroblasts trigger the production of tumor-derived chemokines such as CCL17 and CCL22, which cause infiltration of Tregs. The recruitment of Tregs can also be triggered by fibroblasts with increased MHC-II expression as a result of their interaction with Treg-derived IL1R2. This figure was designed by using graphic elements from BioRender.

Treg cells are one of the major players in driving pro-tumorigenic activity and tumor immune escape, highlighting its potential as a target for cancer therapy (**Figure 5**).

Tregs were initially identified using the CD25 marker. Subsequent studies further revealed the expression of fork-head/winged helix transcription factor (Foxp3). In fact, Tregs function have been shown to be dependent on the active expression of Foxp3, thus conferring Tregs with the ability to play a central role during tumor progression. In addition, expression of Foxp3 has been shown to mediate phenotypic as well as functional conversion of naïve T cells into Treg cells, hence resembling natural Treg cells (63). Therefore, Treg cells are currently identified as a CD4+CD25+FoxP3+ T cell population. The relevance of Tregs in tumor progression is highlighted by recent studies reporting a strong correlation between high infiltration of the TME with FoxP3+ Tregs and poor prognosis in hepatocellular, ovarian, and gastric cancers. However, this observation cannot be generalized to all tumor types given that some cancers, for instance, colorectal cancer, displayed better prognosis with tumors highly infiltrated with high FoxP3+ Tregs (64). In this way, the significance of FoxP3+ Tregs is depends on the biological properties of specific tumor type, stage and molecular subtype.

A novel mechanism that has recently been implicated in Treg accumulation and pro-migratory activity has been shown to be mediated by CD70^+^ CAFs. One study showed that co-culturing CD70-positive CAFs with Tregs resulted in a double fold increase in the population of Tregs. This interaction is particular to Foxp3+ Tregs since no effect has been observed following co-culturing CD70+ CAFs with CD25+ Treg subpopulations. To further highlight the effect of CAF on Treg subpopulations, it was observed that co-culturing CD70^high^ CAFs and CD25 negative Tregs cells, which usually exhibit reduced suppressive abilities, resulted in modulating the phenotype towards a CD25+ Tregs, thus shifting the anti-inflammatory activity of CD25-negative cells to a pro-inflammatory phenotype (65). In addition, other studies show that TGF-β and T cell receptor co-stimulation triggers the expression of FoxP3 gene in CD4+CD25- T cells, contributing to regulatory T cell differentiation into pro-tumorigenic phenotype (66).

In addition to the direct interaction between CAFs and Tregs, an additional mediator has been reported, where tumor cells and CAFs cooperate to recruit Tregs. Indeed, CD68-low CAFs triggered overexpression of chemokines such as CCL17 and CCL22 in tumor cells, which in turn enhanced Treg recruitment. As a matter of fact, infiltration of CD68^low^ CAFs in tumors isolated from patients with oral squamous cell carcinoma correlated with poor survival and more relapse after treatment (67).

Another way of promoting Treg cell infiltration and, hence immune suppression of the TME, is through the expression of interleukin 1 receptor type 2 (IL1R2) by Tregs and its subsequent effect on CAFs. Treg-derived IL1R2 increases major histocompatibility complex II (MHC-II) expression on CAFs, evident from the enhanced expression of MHC-II class genes, which induces Treg infiltration and enforces immune tolerance in TME. In addition, CAF-expressing MHC-II may also suppress CD4+T-cell-induced immune responses. IL1R2 deficient Treg cells cause downregulation of highly expressed genes in several subtypes of Treg cells such as IL10, programmed cell death 1 (Pdcd1), and others, suggesting that IL1R2 is required for activation and clonal expansion of Tregs. Overall, IL1R2 deficiency caused a decrease in Tregs population and infiltration of CD8+ T cells in TME (68).

#### 1.3.4 Macrophages

**Figure 6:**
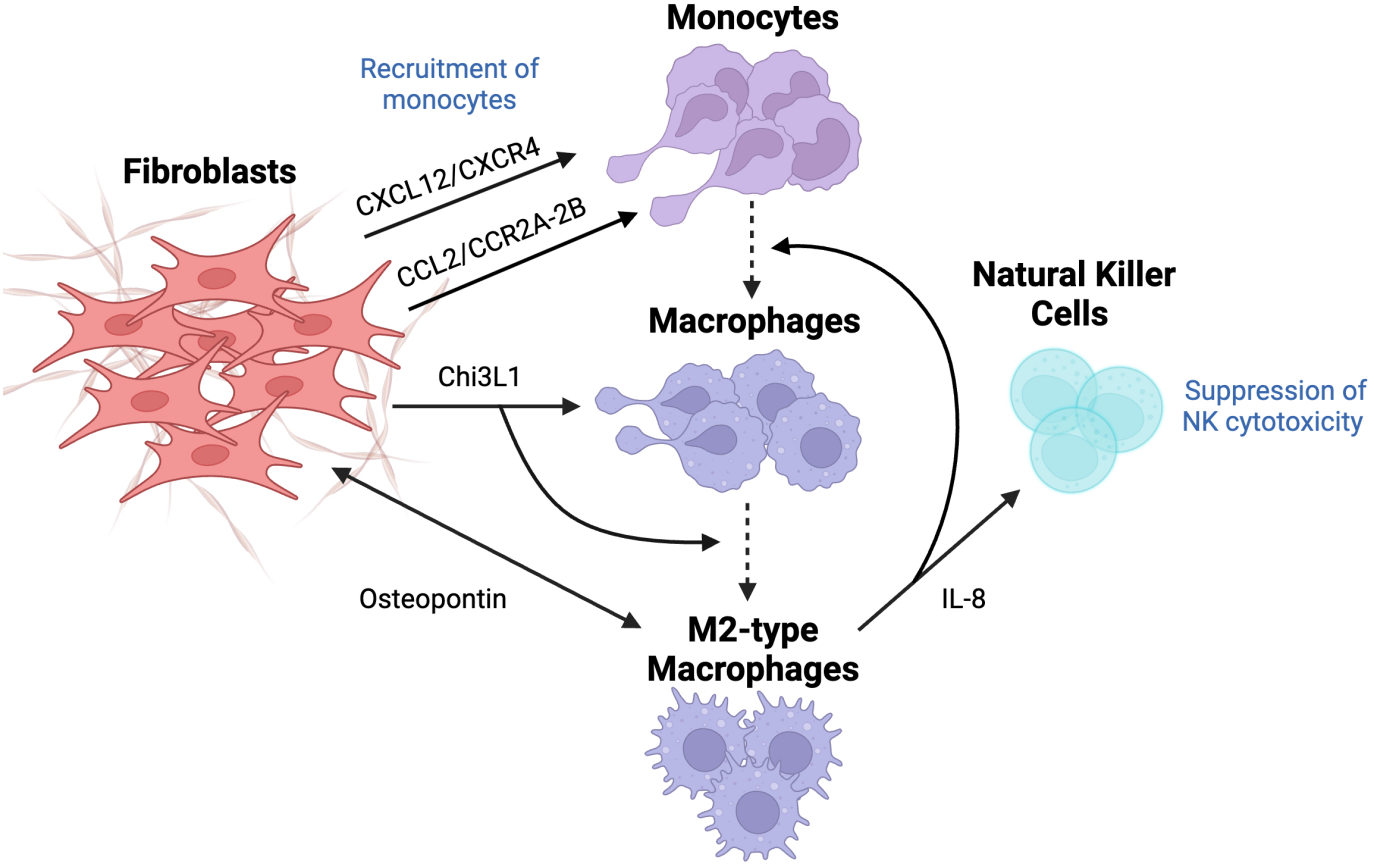
The effect of fibroblasts on the differentiation of macrophages and their tumor-promoting activity. Macrophages usually originate from recruited monocytes, which are recruited through the involvement of CXCL12/CXCR4 and CCL2/CCR2A-2B pathways since CXCL12 and CCL2 are known chemoattractant. Fibroblast-derived chitinase-3 like-protein-1 (Chi3L1), a glycoprotein, results in macrophage infiltration and their differentiation into M2 subtype. Crosstalk between fibroblasts and M2-type macrophages through the secretion of IL-8 causes a further recruitment of monocytes, their differentiation into macrophages, and suppression of NK cytotoxicity. Lastly, fibroblast-derived osteopontin (OPN) activates macrophages, which are, in turn, overexpress OPN to cause fibroblasts release OPN even more, resulting in overall increased levels of OPN. This figure was designed by using graphic elements from BioRender.

Macrophages can be found in almost every tissue, circulating within the body and carrying out different functions. Scientists proposed two macrophage phenotypes: M1 (pro-inflammatory) and M2 (anti-inflammatory). M1 macrophages have a highly phagocytic nature, releasing a considerable amount of reactive nitrogen and oxygen species, in addition to high levels of inflammatory cytokines IL-12 and IL-23, inducing the activation of Th17 cells responsible for inflammation. M1 macrophages usually kill malignant cells through various contact-dependent mechanisms. Interestingly, for the longest time, M1 macrophages were perceived as the only functional phenotype. Currently, it is known that the process is not associated with inhibition of one phenotype but rather polarization of cells into another phenotype, meaning that M1 gets reprogrammed into M2 phenotype. M2, an anti-inflammatory phenotype, contributes to angiogenesis and tissue repair, secreting considerable amounts of IL-10 and other cytokines. Tumor-associated macrophages (TAM), usually perceived as M2 phenotype, were observed to assist in tumor progression through angiogenesis (**Figure 6**) (69).

Since macrophages are mostly derived from monocytes (MO), it is important to elucidate the mechanism of MO recruitment into tumor sites and their differentiation into pro-tumorigenic macrophages. Extensive research was done to explore the recruitment of MOs, specifically the secretion of chemoattractants, suggesting multiple pathways initiated surprisingly by CAFs in different types of cancer. Recent studies highlighted the unique role of the CCL2-CCR2A/2B pathway that attracts MOs in desmoplastic breast carcinoma. CCL2 (MCP-1) is a chemokine overexpressed in CAFs, usually correlated with advanced stages of cancer development and, thus poor prognosis in several types of adenocarcinomas. Interestingly, CCL2-mediated effects are specific to certain subpopulations of MOs. This is highlighted by the inability of CD14+/CD16+ MO to respond to CAF-derived CCL2. Further analysis revealed that the expression of CCR2 on MO is necessary to mediate the crosstalk between MO and CAFs. Subsequent studies reinforced this observation, where blocking CCR2A/2B using specific antibodies completely abolished the recruitment of MO into CAF spheroids, suggesting that CCL2/CCR2 is essential for MO migration. In addition, treatment of MO with pertussis toxin and cholera also suppressed MO recruitment into CAFs. Pertussis toxin targets G-protein function, further confirming the involvement of G-protein in mediating the CCL2 signaling pathway. However, this model of CAF - CD14^+^/CD16^+^ MO communication remains elusive in the presence of many redundant pathways, such as CX3CL1 and CXCL12, which induced MO migration despite the lack of CCR2 expression (70).

Another dominant MO chemoattractant is CXCL12, which mediates its activity through the CXCL12/CXCR4 pathway. Data suggests that CAF-derived CXCL12 recruits MOs that demonstrate M2 macrophage phenotype. Moreover, the blockade of the CXCL12 receptor resulted in decreased chemotaxis of M2 macrophages. The results demonstrated that CAFs cause infiltration of MOs via the CXCL12/CXCR4 pathway followed by their differentiation to M2 phenotype, which implies the potential of CXCL12-mediated macrophage recruitment as a novel target for cancer therapy (71).

In their recent studies, Cohen et al. suggested that CAFs promote immunosuppressive and pro-inflammatory microenvironment in breast cancer by secreting Chitinase 3-like 1 (Chi3L1), a glycoprotein that has been associated with poor survival and prognosis in breast cancer patients. It was found that Chi3L1 is responsible for macrophage recruitment and activation of the M2-like pro-inflammatory gene, in addition to reprograming to M2 macrophage phenotype (72). Moreover, crosstalk between CAFs and tumor-associated macrophages through secretion of IL-8 leads to recruitment and differentiation of MOs, as well as suppression of immune cells. It was observed that the co-culture of CAFs with immune cells induced infiltration and polarization of macrophages, which subsequently led to immunosuppression via impairment of NK cells activity (73), thus highlighting this delicate cross-talk between CAFs and various immune cells within the TME.

Another mediator that was observed in the extensive crosstalk between fibroblasts and macrophages is osteopontin (OPN), a key chemokine-like glycophosphoprotein that is responsible for many tumor-promoting processes in TME. OPN plays a role in cellular signaling and cell-matrix interactions, regulating cytokine production, cell migration and adhesion, majorly contributing to cancer progression. The key findings highlight that OPN is secreted only from CAFs activated by TAM, reflecting heterogeneity in the origin of CAFs. Macrophages-derived CAFs showed increased expression of αSMA but lower expression of IL-6, which can be explained by the fact that functionality may differ due to only CAF-like phenotype and their origin. In fact, overexpression of OPN in macrophages has been shown to increase the secretion of OPN by CAFs, leading to overall enhanced cancer malignancy. This suggests that OPN inhibitors may block and abolish cancer-TAM-CAF interactions, leading to decreased cancer malignancy (74).

#### 1.3.5 Myeloid-derived suppressor cells

**Figure 7:**
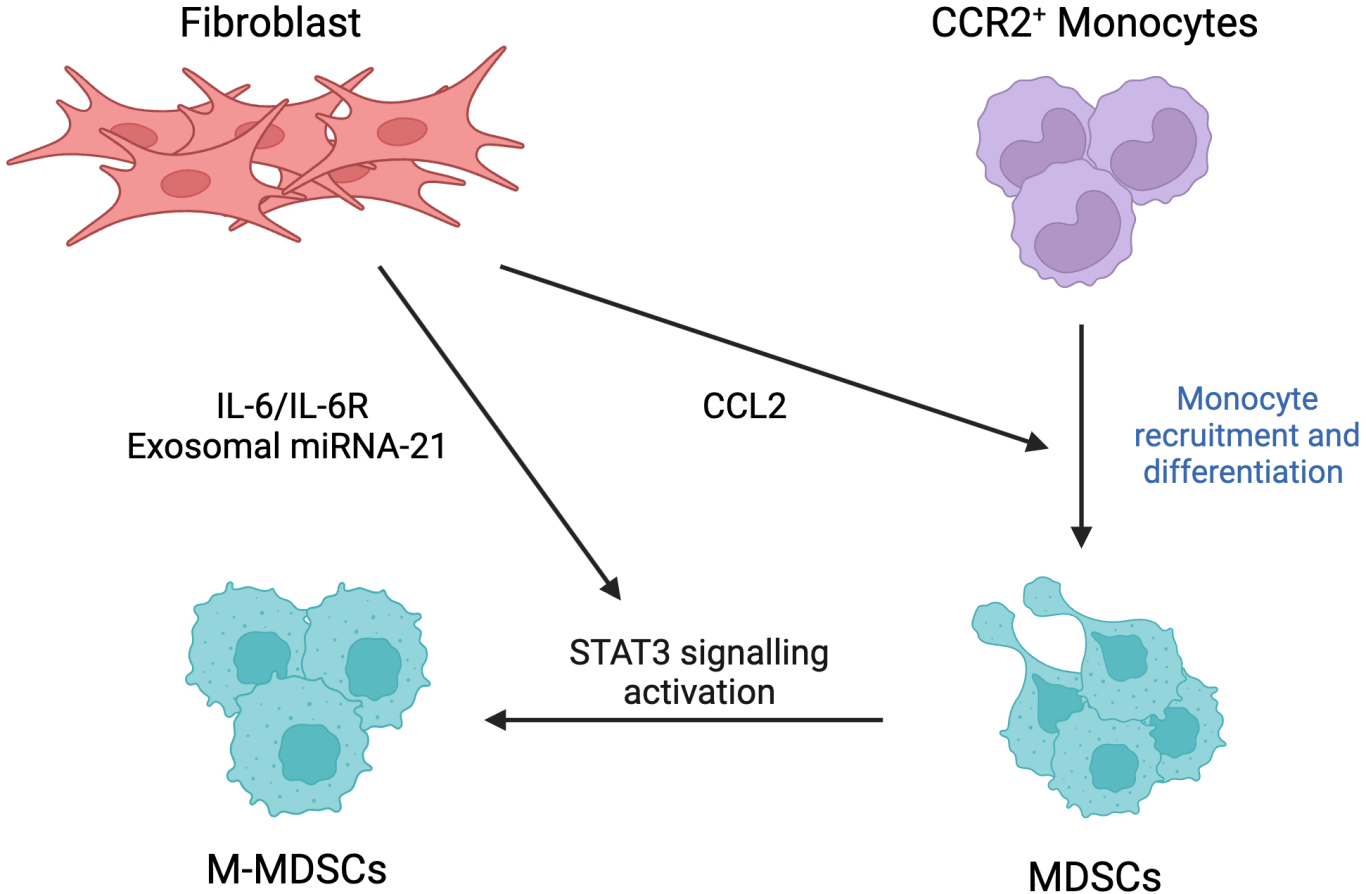
Fibroblast-driven recruitment and differentiation of myeloid-derived suppressor cells (MDSCs). Fibroblasts promote recruitment of CCR2-positive monocytes through the release of CCL2, further inducing their differentiation. Fibroblasts induce activation of STAT3 signaling pathway, responsible for generation of monocytic-MDSCs (M-MDSCs), via triggering IL-6/IL-6R pathway in a paracrine and autocrine manner and fibroblast-derived exosomal miRNA-21. This figure was designed by using graphic elements from BioRender.

Originating from bone marrow, MDSCs are a group of myeloid cells distinct from already differentiated mature myeloid cells such as dendritic cells, neutrophils, and macrophages. MDSCs include a group of various myeloid progenitors along with immature mononuclear (75). This population consists of two main subtypes, which include mononuclear (M-MDSC) and polymorphonuclear (P-MDSC) that are phenotypically and morphologically similar to monocytes and neutrophils, respectively (76). As their name suggests, these progenitor cells play an important role in immune suppression of antigen-specific and nonspecific T cells, mainly by releasing NO and cytokines, thus suggesting its potential role in promoting cancer (**Figure 7**). Once recruited into the tumor site, immature MDSCs are differentiated into mature myeloid cells, further contributing to immunosuppression (75).

Through activation of STAT3 signaling pathways, FAP^+^CAFs have been recently shown to mediate the major infiltration of MDSCs, which subsequently resulted in suppression of IFNγ+T-cells. Indeed, FAP activates inflammatory CAFs by triggering STAT3, which is an important transcription factor involved in tumor-associated inflammation. In pathological conditions similar to tumor progression, such as skin fibrosis, FAK is a major effector molecule that is activated downstream of the STAT3 pathway, and it has been shown to correlate with activation of the ERK-CCL2 signaling pathway. Interestingly, using a murine liver tumor model, c-Src was found to be a major downstream effector rather than ERK, which results in enhanced expression of CCL2. It promoted the recruitment of MDSCs, resulting in a more immunosuppressive TME to abolish the activity of anti-tumor IFNγ+T-cell immunity (28).

Recent studies point to the role of CAFs as the culprits in mediating drug resistance, and this is largely attributed to their close cooperation with the MDSCs, hence providing a new potential therapeutic target. CAFs induce the generation of M-MDSCs via activation of the STAT3 signaling pathway through IL-6/IL-6R in a paracrine and autocrine manner. The fact that inhibition of STAT3 completely abolished the production of CAF-derived MDSCs further supports the role of STAT3 in mediating the interplay between CAFs and recruited MDSCs. On the other hand, blocking IL-6 resulted only in partial inhibition of the generation of MSDCS, suggesting an involvement of other mechanisms/pathways. Indeed, recent studies indicate that STAT3 can also be activated by exosomal miRNA-21 released by CAFs and transferred to MOs to target PTEN in the cytoplasm. It is suggested that PTEN targeted by miRNA-21 may activate STAT3 through the mTOR pathway (77).

MSDCs also exert immunosuppressive activity by abolishing anti-tumor T-cell immunity. CAFs play a main role in the activation of MDSCs as they promote CCR2+ MO migration through secretion of CCL2, followed by their differentiation into MSDCs. MDSCs, in turn, drive immunosuppression through the expression of 2,3-dioxygenase-1 (IDO1), which is responsible for inflammatory programming and MDSC support, and protein complex NOX2, which produces excessive amounts of ROS. Secretion of ROS results in T-cell apoptosis, which is further supported by the observation of restored T-cell proliferation following treatment with NOX2 inhibitors (78).

In a novel mechanism, it was found that myofibroblasts may also have tumor-restraining activity, which is mediated by the expression of type I collagen. Using single-cell RNA sequencing, myofibroblasts were identified as the major cells that mostly contribute to the expression of large amounts of type I collagen within the TME. Furthermore, type I collagen expression was observed to colocalize with αSMA+ myofibroblasts. Interestingly, the knockdown of type I collagen results in increased infiltration of MDSCs that secrete high levels of CD206, F4/80, CCL2 and IL-18, which subsequently result in suppression of T and B cells. Regarding the mechanisms by which CAF-derived type I collagen mediate its effects, it is suggested that a decrease in levels of Col1 within the tumor stroma may trigger the expression of CXCL5 via Sox9 expression, which contributes to the recruitment of MDSCs (3).

#### 1.3.6 Cytotoxic T cells

**Figure 8:**
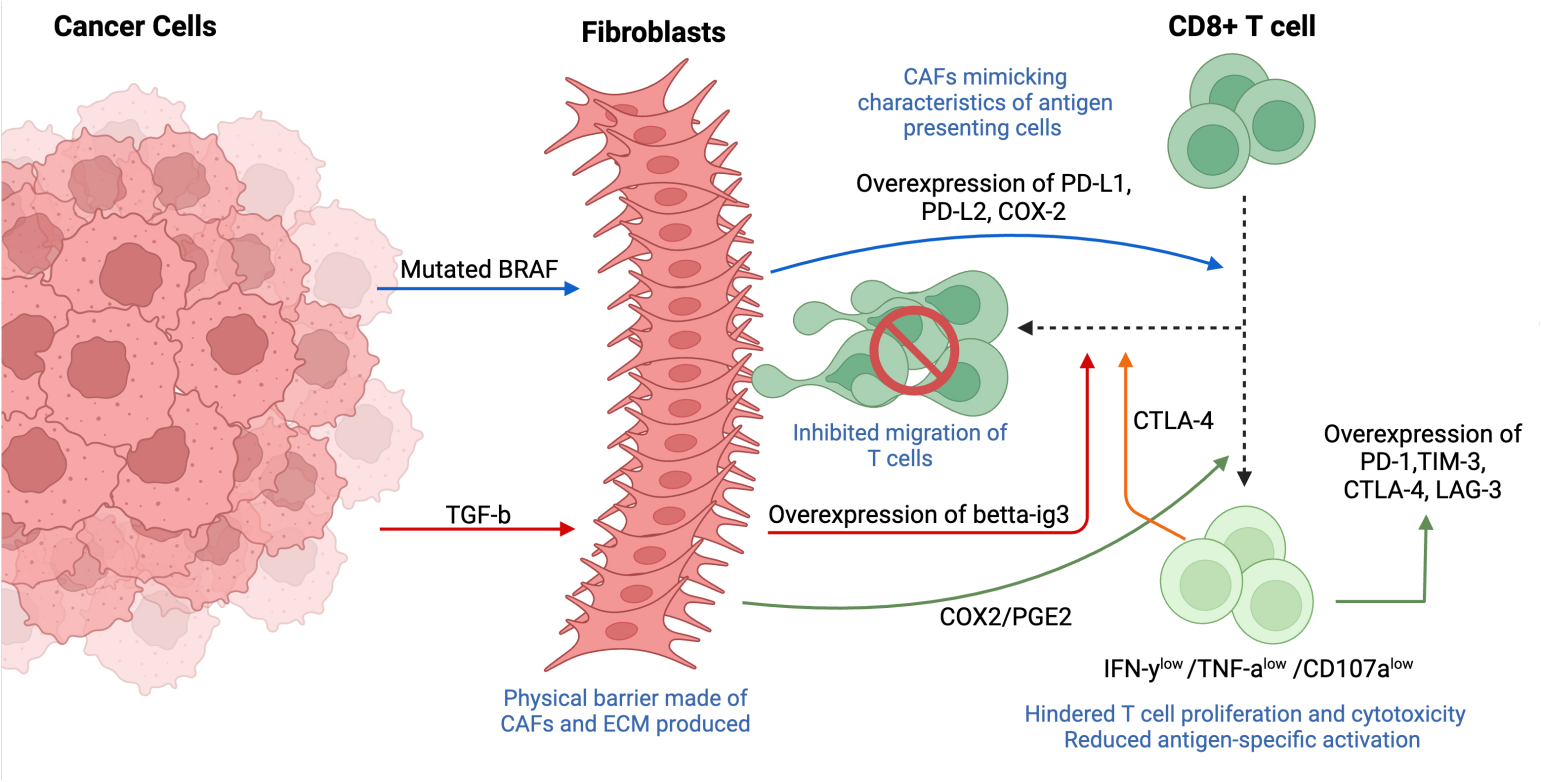
Tumor- and fibroblast-induced suppression of cytotoxic T cells (CD8+ T cells). The immunosuppression of T cells is mediated through multiple mechanisms such as downregulating MHC-1 and/or physically restricting the cells from the tumor mass. Fibroblasts usually localized around tumor cells, forming a physical barrier between CD8+ T cells and tumor. Tumor-derived mutated BRAF activates fibroblasts, causing an overexpression of CAF-derived PD-1 ligands (PD-L1, PD-L2, COX-2), thus mimicking characteristics like antigen-presenting cells and resulting in T cell exhaustion. Fibroblasts induce the expression of inhibitory markers (TIM-3, PD-1, CTLA-4, LAG-3) on cytotoxic T cells through PGE-2, a major metabolite of COX2. Overexpression of fibroblast-derived betta-ig3 caused by expression of cancer-derived TGF-β results in increased fiber thickness and reduced migration of T cells. T cell-derived CTLA-4 overexpressed in CAF-rich tumors enhances T cell adhesion, also, contributing to decreased motility of CD8+ T cells. This figure was designed by using graphic elements from BioRender.

CD8+ T cells or cytotoxic T cells are a major part of the adaptive immune system that interact with MHC class-1 (MHC-1) molecules on the surface of target cells. Once T cells recognize and interact with a target cell, they enhance pore formation in its membrane and secrete granules consisting of granzymes, perforin and granulysin. However, tumor cells mediate the immunosuppression of T cells and activate defense mechanisms that may downregulate MHC-1 or keep T cells at a distance from tumor cells because their activation requires the physical interaction between T cell and tumor cells (**Figure 8**) (79). To suppress anti-tumor T cell immunity, CAFs produce numerous soluble factors to sequester the migration of T cells into the tumor islets and locate them around the tumor mass (80).

The potential role of CAFs in immune cell suppression has been shown *in vivo* by the localization of CAFs within TME and prominent expression of certain soluble factors, including T-cell-suppressive soluble factors, in addition to the engagement of immune checkpoints (81). Indeed, scientists found that mutated BRAF, a gene responsible for the regulation of IL-a and -b transcription in tumor cells, induced the activation of CAFs in melanoma, which resulted in the enhanced expression of CAF-derived programmed death 1 (PD-1) ligands such as programmed death-ligand 1 (PD-L1), PD-L2 and cyclooxygenase 2 (COX-2). The secretion of PD-1 ligands by CAFs demonstrates a new biological function for fibroblasts that contributes to immunosuppression by mimicking characteristics that are reminiscent of antigen-presenting cells (82). The involvement of BRAF in T cell exhaustion was further supported by its inhibition and following significant reestablishment of anti-tumor T cell responses, implying the potential role of BRAF inhibitors in immune-based therapies (83).

Interestingly, CAFs induced the expression of inhibitory markers/immune checkpoint molecules TIM-3, PD-1, cytotoxic T-lymphocyte associated protein 4 (CTLA-4) and lymphocyte-activation gene 3 (LAG-3) in proliferating CD8+ T cells, which induced differentiation of T cells into exhausted phenotype associated with decreased proliferation and suppressed cytotoxic ability. These findings also revealed that CAFs mediate their immunosuppressive abilities by binding CAF-derived PD-L1/L2 ligands to the inhibitory PD-1. A new player, COX2, and more specifically, its major metabolite – PGE2, has recently been highlighted to mediate T cell proliferation and induce overexpression of PD-1 and TIM-3. Additional functional assays demonstrated a decreased production of inflammatory cytokines IFN-γ and TNFα, in addition to degranulation marker CD107a in T cells after restimulation in the presence of CAFs, indicating their potential involvement in a weakened immune function. More importantly, both studies mentioned above stressed the localization of CAFs within the architecture of TME, frequently forming a physical barrier between T cells and tumor cells, which implies that CAFs are strategically located in the stroma to induce and maintain immunosuppression (81, 83).

Cytotoxic T cells exhibit a migratory phenotype, allowing them to change their morphology and even deform the nucleus to move through the matrix. The protease-independent movement of T cells depends on the surrounding matrix density, thus forcing them to follow the least resistant pathways (84). By physically restricting T cells from the tumor mass and controlling their infiltration through production of ECM components, CAFs may influence tumor response to immune therapy (84). Indeed, tumors with CAF predominance are often associated with desmoplasmic stroma that contains collagen, fibronectin, and various proteoglycans, which limit physical contact between T cells and tumors. The role of CTLA4, one of the most overexpressed genes in T cells isolated from CAF-rich tumors, has been recently explored. It was observed that CTLA-4 enhances T cell adhesion and decreases its motility by mediating integrin activation. Indeed, introducing anti-CTLA-4 antibodies resulted in restoring CD8+ T cell tumor infiltration and motility (85). Similar results were observed in another study, where anti-CTLA4 significantly accelerated T cell motility within the tumor (86).

Beta-ig-h3 (also called TGFBI), an ECM protein involved in cell-matrix interactions and cell migration, has recently been indicated to directly reduce T cell immunity by densely accumulating in the stroma and reducing antigen-specific activation. Moreover, patients with a densely accumulated big-h3 expression, not in tumor cells but rather in the stroma, had a poor prognosis. Further studies provided more mechanistic insights into the association of beta-ig-h3 and CAFs. As expected, depletion of beta-ig-h3 resulted in reduced fiber thickness, which is usually associated with better prognosis in patients with pancreatic ductal adenocarcinoma (PDA) (87). Both studies indicate the importance of targeting CTLA-4 and/or depleting beta-ig-h3 and other ECM proteins to resensitize CAF-rich tumors to immunotherapy, in addition to restoring T cell proliferation and cytotoxicity.

#### 1.3.7 Dendritic cells

**Figure 9:**
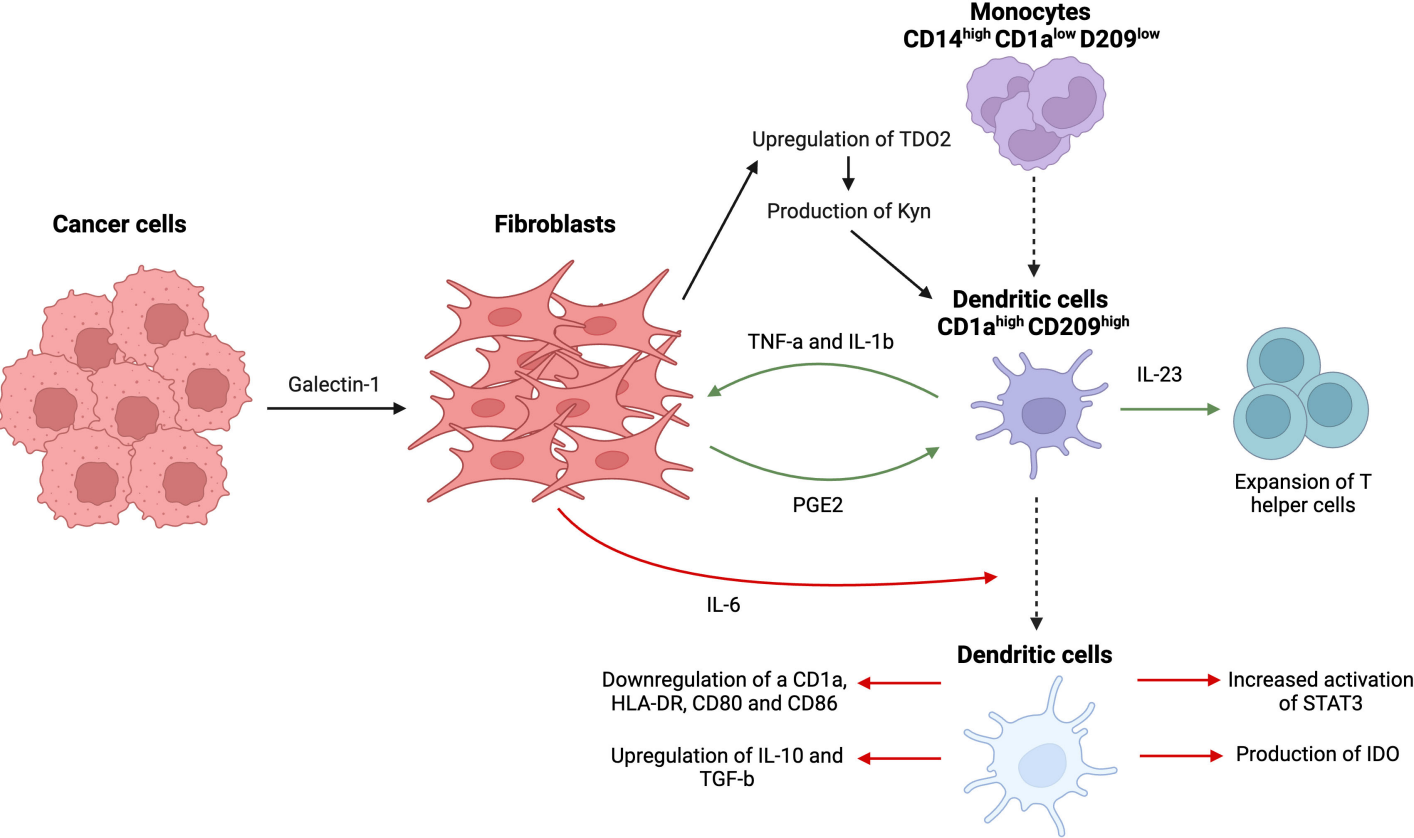
Complex interactions between tumor cells, dendritic cells, and fibroblasts, resulting in prevalence of an immunosuppressive tumor microenvironment. Tumor cell-derived galectin-1 causes an upregulation of TDO2, the main enzyme in tryptophan metabolism in CAFs, and promotes disproportionate production of kynurenine (Kyn), a tryptophan metabolite. Kyn suppresses activity of DCs and their differentiation from monocytes. Fibroblasts themselves release IL-6, causing decrease in antigen-presenting receptors (CD1a, HLA-DR, CD80, and CD86), overexpression of immunosuppressive cytokines (IL-10, TGF-β), upregulation of STAT3 phosphorylation, and production of indoleamine 2,3-dioxygenase (IDO). A delicate feedback loop that involves DC-derived TNFα/IL-1b and CAF-derived PGE2 leads to activation of fibroblasts and overexpression of IL-23 responsible for Th17 cells expansion. This figure was designed by using graphic elements from BioRender.

Dendritic cells (DCs) are bone marrow-derived antigen-presenting cells, which establish a link between innate and adaptive immune responses by capturing and processing antigens (88). They are involved in the activation of naïve T cells via transforming proteins to peptides presented on MHC molecules. DCs that have been educated by the tumor or CAFs maintain an immunosuppressive microenvironment by decreasing the infiltration of cytotoxic T cells and increasing the migration of suppressive Tregs (**Figure 9**).

CAFs have been shown to negatively affect MO differentiation into DCs in addition to modulating functional features of DCs such as antigen uptake, expression of stimulatory molecules, and infiltration of other lymphocytes such as T cells. For example, CAF-derived IL-6 caused decreased expression of antigen-presenting receptors such as CD1a, human leukocyte antigen-DR isotype (HLA-DR), CD80 and CD86, and overexpression of immunosuppressive cytokines including IL-10 and TGF-β using a hepatocellular carcinoma model (89). Interestingly, IL-6 derived from CAFs-induced upregulated phosphorylation of STAT3 in DCs, a surprising result given the previously similar results reported for IL-6 derived from tumor cells. Indeed, treatment with IL-6 resulted in increased activation of STAT3 and production of indoleamine 2,3-dioxygenase (IDO), responsible for suppressing T cell immunity (90). An alternative pathway of inhibition of MO differentiation into DCs includes upregulated expression of CD14 (MO marker) and decreased levels of DC signature markers such as CD1a and CD209, which are essential for DCs antigen uptake capacity (89, 91).

Tumor cells may also indirectly participate in the process by metabolically reprogramming CAFs through tryptophan catabolism. This crosstalk between CAFs and cancer cells involves a novel process called metabolic coupling, resulting in a disproportion of the tryptophan metabolite kynurenine (Kyn), which is responsible for hampering the process of differentiation of dendritic cells. High levels of Kyn are usually associated with poor prognosis and enhanced cancer progression (92). It was observed that lung cancer-derived galectin-1 results in the upregulation of tryptophan 2,3-dioxygenase (TDO2), the main enzyme involved in tryptophan metabolism in CAFs, via an Akt-dependent pathway, promoting the production of Kyn by CAFs that further suppresses the activity of dendritic cells (92). Similar to previous studies mentioned above, DCs produced in the presence of lung cancer-derived CAFs failed to downregulate monocytic markers and to upregulate dendritic markers, suggesting failed differentiation and dysfunction of DCs, rendering them unable to activate T cell immunity. This was further supported by the knockdown of TDO2 that led to restored anti-tumor T cell immunity through the activation of DCs, substantiating the role of CAF and cancer-derived Kyn in the immunosuppression (91).

A recent study reported a rather novel mechanism downstream of the crosstalk between fibroblasts and dendritic cells, which is the expansion of T helper cells (Th17). Under inflammatory conditions, DCs release high levels of pro-inflammatory TNF-a and IL-1b, further activating resident fibroblasts. This stimulation of fibroblasts led to a secretion of prostaglandin E2 (PGE2), which in turn induced an overexpression of IL-23 in dendritic cells that was responsible for a significant expansion of Th17 cells from the memory pool of T cells. Hence, this delicate interaction involves a complex feedback loop that involves DC-derived TNFα/IL-1b and CAF-derived PGE2, highlighting the crucial role played by CAFs in modulating the functionality of DCs and, subsequently, the polarization of the T cell response (93).

## 2 Discussion: therapeutical potential

### 2.1 Macrophages

**Figure 10:**
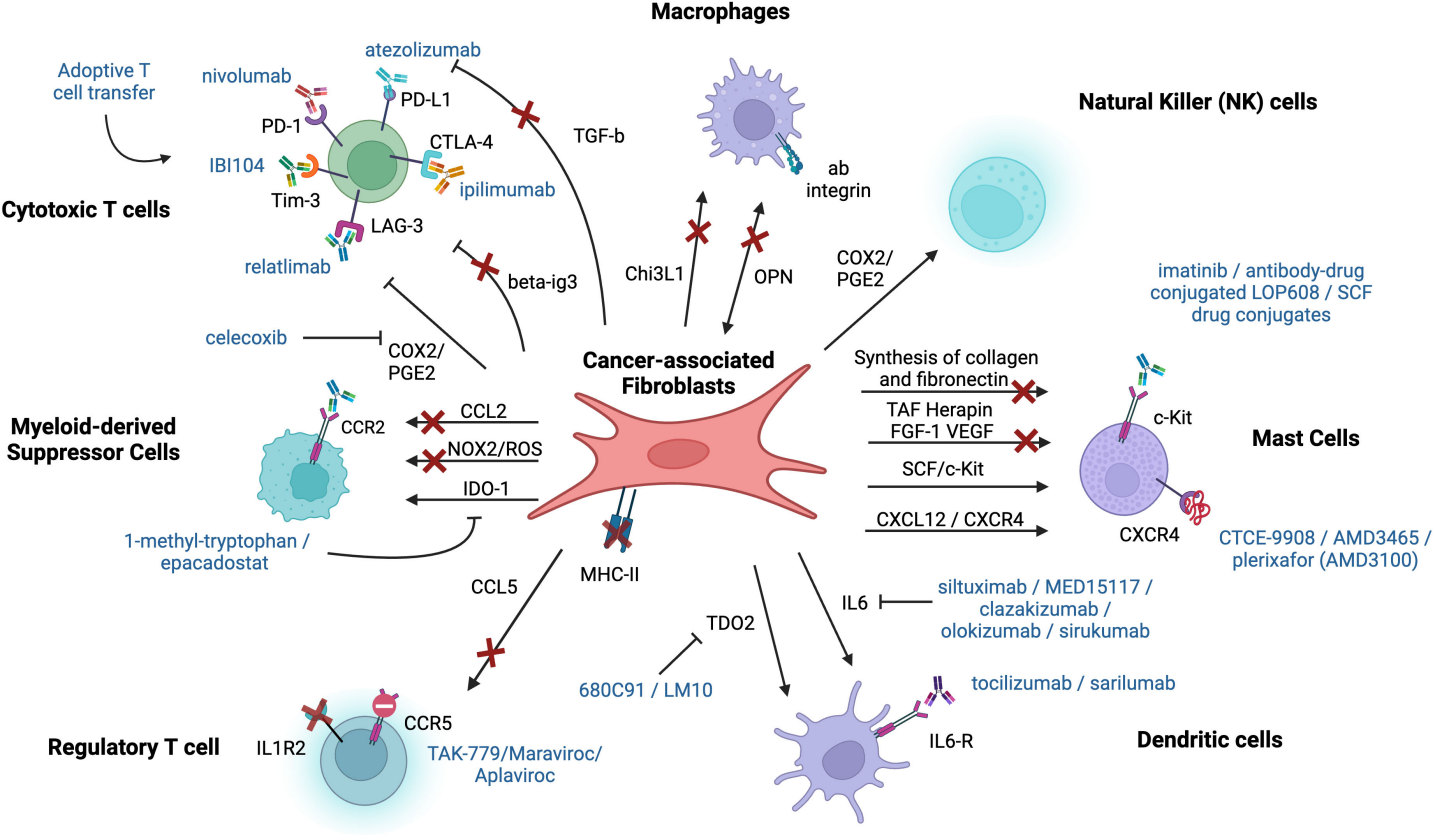
Therapeutic potential behind the crosstalk and interactions between immune cells and fibroblasts. Each arrow represents a pathway or an interaction between fibroblasts and immune cells that can be a potential target for cancer therapy. Already existing therapeutic agents are shown near the corresponding receptor or interaction (in blue). The arrows with the red cross represent pathways or interactions that should be further researched and considered as a potential target for immunotherapy. This figure was designed by using graphic elements from BioRender.

In the context of crosstalk between fibroblasts and macrophages, the recruitment of macrophages takes place through the interaction between OPN and alpha4 and alpha9 integrins, suggesting that targeting CAF-derived OPN expression can reduce macrophage infiltration into the TME (**Figure 10**). Although OPN can also be expressed by tumor cells, fibroblasts represent a more desirable therapeutic target since they are more genetically stable. There are multiple OPN-targeting preclinical therapeutic approaches, including gene silencing, receptor blockage, or posttranslational modification, that had been proven to be effective in cancer treatment. Ongoing research suggests that RNA interference-meditated inhibition of OPN using microRNA, siRNA, and shRNA induces a tumor-suppressive effect (Liu S., et al., 2008). However, the main challenge associated with this method is non-selective inhibition of both the secreted and intracellular forms of OPN, which have opposing effects on cancer progression (94). Using antibodies that block integrins is another way of targeting OPN. Indeed, the blockade of avb3 and a9b1 integrins resulted in attenuation of tumor growth and angiogenesis using a breast cancer model. However, it might be challenging to design antibodies against each integrin that has been shown to play a role in the OPN- mediated interactions. In addition, scientists also explored the potential of small molecule protein-protein interaction (PPI) inhibitors, specifically the efficiency of a novel inhibitor, IPS-02001, in blocking integrin signaling. The inhibitor showed a suppression of downstream signaling of integrin a_v_b_3_ in osteoclasts (OCs) due to high expression of the integrin and its role in facilitating the attachment of OCs to bone (95). We would like to highlight the fact this new inhibitor has not yet been tested for cancer treatment hence it has the potential to be a breakthrough therapy. OPN stands as a compelling prospect for cancer therapy, given its role in cancer progression and possible ways of blocking its mediated effects. However, it shares limitations similar to other immunotherapies, including resistance, redundancy, and side effects due to its pleiotropic activities.

CAF-derived Chi3L1 was observed to be another signaling axis between fibroblasts and immune cells, resulting in immunosuppressive and tumor-promoting microenvironment (**Figure 10**). Studies suggest that CAF-derived Chi3L1 causes a shift from type 1 to type 2 immunity characterized by overexpression of factors associated with inflammation, such as IL-10, IL-14, and IL-13, and reduced IFNy and TNFα synthesis. In contrast, the knockdown of Chi3L1 results in enhanced type 1 immunity and increased T cell infiltration (72). The role of Chi3L1 was also studied in a non-cancer context, such as fibrosis, where scientists observed its role in profibrotic macrophage activation, essential for progression of pathologic fibrosis, and fibroblast differentiation (96).

### 2.2 Dendritic cells

IL-6 is secreted by various cell types in TME, including immune cells, stromal cells, and cancer cells, inducing the activation of Janus kinase (JAK)/STAT3 signaling, which in turn drives immunosuppression. It exerts negative regulatory effect on dendritic cells, not only resulting in their exhaustion but also hampering their differentiation from MOs. IL-6 levels usually strongly correlate with tumor size, stage, and progression of cancer. Scientists came up with three main ways of hindering IL-6 signaling by direct targeting of IL-6 and IL-6R with antibodies, as well as selectively inhibiting the IL-6-IL-6R complex using fusion proteins (**Figure 10**) (97).

Siltuximab, a chimeric antibody constructed using domains of mouse and human, is the leading clinical agent targeting IL-6 that was Food and Drug Administration (FDA) approved in 2014. Although preclinical trials have demonstrated promising outcomes, clinical trials have provided limited evidence regarding its efficacy against solid tumors, emphasizing the need to further elucidate its mode of action and the necessity for development of combination therapies. There are additional antibodies targeting IL-6, which are currently undergoing preclinical trials in cancer, such as MED15117, clazakizumab, olokizumab, and sirukumab (98). In addition, there is another FDA approved treatment for patients with arthritis and B cell acute lymphoblastic leukemia under CAR-T therapy, Tocilizumab, which recognizes IL-6R and disrupts the signaling cascade. Tocilizumab is a humanized monoclonal antibody that showed promising results in preclinical studies. Scientists are exploring its potential in B cell chronic lymphocytic leukemia, breast, and pancreatic cancer. Sarilumab is another monoclonal study that is in preclinical development (98). It is important to consider that there are other activators of JAK/STAT3 pathway besides IL-6, and only individual nodes of the target, including IL-6 and IL-6R, are targeted by FDA approved agents, not necessarily in the context of cancer. There is a clear need in novel inhibitors and combination therapies that will show higher efficiency in both preclinical and clinical studies.

To hamper the differentiation of MOs into dendritic cells, cancer cells can cause changes in the way that CAFs metabolize tryptophan. Cancer cell-derived galectin 1 causes overexpression of TDO2 that induces enhanced synthesis of Kyn, a suppressor of dendritic activity. TDO2 has been identified in various cancer types, including malignant glioma, ovarian carcinoma, breast cancer, and melanoma. Originally, TDO2 inhibitors were created to serve as antidepressants because elevated levels of tryptophan boost serotonin level in the brain. Currently, there is ongoing development of TDO2 small-molecule inhibitors for cancer treatment, which have not yet progressed to clinical trials (**Figure 10**). Inhibitors, such as 680C91, LM10, and a few patents by RedXpharma and IOmet Pharma, are under experimental or preclinical studies (92). There are other tryptophan-catabolizing enzymes, including IDO1 and IDO2, which play a role similar to that of TDO2, emphasizing the importance of focusing on targeting other enzymes involved in tryptophan metabolism (99). Due to the lack of success in recent clinical trials regarding the inhibition of the tryptophan-catabolizing enzymes, there is a possibility that pharmaceutical companies might discontinue to pursue the idea of inhibiting these enzymes (92). Moreover, there are alternative approaches that might be used in combination with TDO2, such as targeting galectin released by cancer cells, which could activate CAFs. However, one of the challenges in developing galactin-1 inhibitors is to make them highly specific and potent while minimizing unwanted side effects. Another challenge is to achieve promising results in preclinical trials (100).

### 2.3 Mast cells

KIT belongs to the type III receptor tyrosine kinase family, which usually acquires gain-of-function mutation, resulting in constitutive phosphorylation and activation of the cytoplasmic domain independent of stem cell factor (SCF). KIT mutation is found in 80% of gastrointestinal stromal tumors. One of the most interesting FDA-approved kinase inhibitors that targets mutated domains of KIT and achieving a disease control in 80% of advanced patients is Imatinib (**Figure 10**). However, imatinib becomes ineffective once tumor accumulates secondary mutations of KIT. In addition, there are some novel therapy methods such as antibody-drug conjugate (ADC) LOP608 that takes advantage of antibody specificity and high toxicity of the conjugated drug. ADC NN2102-DM1 was observed to be efficient in KIT-expressing cancer in mouse models, which offers another promising way of targeting both mutant and wild-type KIT (101). However, these studies were done on cancer cells, but more research is needed to see if the results also apply to fibroblasts.

Another exciting target is SCF, which is the ligand of KIT (**Figure 10**). Due to its ability to recycle and mediate ubiquitylation of KIT, SCF is implied in regulation of SCF/KIT signaling. Interestingly, mutant KIT has the ability to autophosphorylate, independent of SCF. However, SCF is still able to bind to mutant KIT and induce its internalization, which makes it a potential vector to deliver drugs into cancer cells. Hence, researchers tested a drug conjugate, SCF-DM1, in gastrointestinal stromal tumor (GIST) cells, revealing inhibitory effect on KIT signaling. This suggests that the ligand of KIT, SCF, could also be used as the antibody in conjugated drugs (101).

CXCL12/CXCR4 axis is also responsible for mast cells recruitment from normal vasculature or healthy tissues, which turns it into a potential target for cancer therapies (**Figure 10**). Researchers introduced multiple CXCL12/CXCR4 inhibitors and antagonists to observe its effect on tumor development. CTCE-9908 is a small peptide analog of CXCL12, exerting antagonist activity on CXCR4. It was observed that introduction of the analog to osteosarcoma cells *in vitro* led to reduced adhesion, migration, and growth rate. Similar effects were reported in other cancers like ovarian cancer and breast cancer using mouse model systems. A promising drug called AMD3465, is currently the focus of preclinical studies. This drug blocks CXCR4 and opposes CXCL12 stimulation of chemotaxis and cell proliferation pathways in leukemia cells (102).

Among all other antagonists, plerixafor (AMD3100) is the FDA-approved CXCR4 antagonist that blocks the interaction between CXCL12 and CXCR4 (**Figure 10**). Interestingly, a phase 1/2 demonstrated that combination of plerixafor with cytotoxic chemotherapy might increase the remission rate in acute myeloid leukemia. In addition, plerixafor in combination with radiochemotherapy led to decreased tumor growth and lymph node metastasis in cervical cancer compared to monotherapy. Furthermore, treatment with AMD3100 in mice with ovarian cancer showed significant rise in antitumor immune responses mediated by T cells and resulted in tumor apoptosis and necrosis (102). However, scientists are still proposing more efficient antagonists such as MSX-122, which is one of the most potent CXCR4 inhibitors carried forward as a clinical drug candidate. Due to its chelating ability, AMD3100 can contribute to cardiotoxicity and increased mortality observed in mice with pulmonary fibrosis. In contrast, MSX-122 inhibits CXCR4 without metal-chelating capability, which increases its effectiveness compared to AMD3100 and emphasizes the need to develop safer and more effective drugs with fewer side effects (103).

Lastly, a recent study emphasized the role of FAP-positive CAFs and their expression of CXCL12 that resulted in no response to two immune checkpoints, CTLA-4 and PD-L1. They observed immune control only after depleting FAP+CAFs, which were responsible for overexpression of CXCL12 that restricted recruitment of T cells into the tumor site. CXCR4 inhibitor led to rapid T cell infiltration and acted synergistically with PD-L1 (104).

### 2.4 Myeloid-derived suppressor cells

Considering the role of CCL2 in MDSC recruitment and fostering a tumor permissive environment, CCl2 blockade would potentiate immune response and augment immunotherapy (**Figure 10**). Application of CCL2 antagonists resulted in decreased MDSC infiltration and inhibited tumor development. In addition, its combination with anti-PD1 antibody was observed to increase recruitment of both CD4+ and CD8+ cells along with IFNγ production, which is usually impaired in tumors due to infiltration of MDSC. Development of resistance to PD-1/PD-L1 inhibitors develops due to secretion of CCL2, which highlights the importance of combination therapy to potentiate immune response to PD-L1 blockade (105). CCR2, the CCL2 receptor, represents another way of targeting CCL2 release, that in combination with anti-PD-1 therapy resulted in sensitization and increased tumor response compared to monotherapy. This approach serves as strong preclinical rationale to further explore CCR2 antagonism and its combination with other therapies, including immune checkpoint blockade (106).

IDO-1 protein is the next potential target for mediating immunosuppressive effects in cancer and expressed by numerous stromal cells, including fibroblasts. Its blockade was achieved through its pharmacological inhibitor, 1-methyl-tryptophan (1MT), and clinically tested using the competitive inhibitor, epacadostat. Combination of pharmacological inhibition and anti-CTLA-4 antibody led to rejection of established tumors and resistance to secondary challenge in mice after inoculation with melanoma cells, which was mainly achieved through restored CD8+ and CD4+ T cells activity. On the other hand, epacadostat was tested in phase 1/2 clinical trials resulting in near maximal inhibition achieved. The outcome of the treatment was assessed based on Kyn/Trp baseline value in melanoma patients, a method used to quantify Trp and Kyn in plasma/serum where increased baseline value represents enhanced IDO activity and is usually associated with advanced disease stages. Interestingly, the increase in the ratio was independent of anti-CTLA-4 treatment. However, in a phase 3 trial epacadostat showed limited success when used in combination therapy with anti-PD-1 treatment (107). Some believe that it might be due to utilization of Kyn/Trp as an assessment of inhibitor’s efficacy since its concentrations range in the cancer and it is challenging to accurately deduce it. Moreover, it is suggested that mechanism associated with the IDO signaling pathway is more likely to converge on other pathways, such as ROS generations, as well as IDO2 and TDO (78). For this reason, it is important to elucidate intricate web of pathways and mechanisms to be able to manipulate immune climate in TME using interactions between fibroblasts and MDSCs.

It is known that ROS production significantly affects T cells and their proliferation. It was found that inhibition of NOX2, a protein complex responsible for ROS production, results in decreased ROS secretion in CAF-educated MDSCs, recovering antitumor T cell immunity (**Figure 10**) (78).

### 2.5 CD8+ T cells

In addition to the ability of CAFs to impede the migration of T cells into the TME, suppression of tumor infiltrating cytotoxic activity of CD8+ T cells may be also achieved through the mechanisms that include CAF- derived ligands or ECM components. One approach to counteract CAF-mediated immunosuppression of cytotoxic T cells may be accomplished through numerous inhibitors that target these pathways (**Figure 10**). For instance, inhibiting PD-L1 pathway using atezolizumab (anti-PD-L1 agent) has been shown to induce a robust and lasting effect but only in a subset of cancer patients. The lack of response was prevalent in patient-derived tumors that were characterized by the specific localization of CD8+ T cells to peritumoral areas that were predominantly rich in fibroblasts and collagen. Furthermore, fibroblasts isolated from these tumors displayed a specific signature of TGF-β signaling. Therefore, the efficacy of targeting TGF-β signaling pathway in combination to using anti-PD-L1 antibodies was explored in various mouse tumor models. Interestingly, co-inhibition of TGF-β and PD-L1 signaling pathways resulted in less physically restricted and more mobile T cell phenotype (108). Additional immune checkpoint approaches were also targeted by monoclonal antibodies, mainly in combination with PD-1 inhibitors. Ipilimumab is considered the first FDA approved anti-CTLA4 monoclonal antibody used in treatment of melanoma. Tumors with high mutation load were observed to be more responsive to a combination treatment of ipilimumab and nivolumab (anti-PD-1-agent). Co-inhibition has been shown to enhance the proliferative rates of cytotoxic and regulatory T cells as well as cytokine release (109). Similarly, PD-L1 pathway was also targeted along with Tim-3 and LAG-3 pathways by administering IBI104 and relatlimab, respectively (110, 111). Indeed, a combination therapy targeting Tim-3 and PD-L1 resulted in a significant reduction in tumor growth. Nonetheless, 50% of the mice experienced complete tumor regression. Similar results were observed in chronic viral infection, where simultaneous targeting of Tim-3 and PD-L1 resulted in a synergistically improved response of cytotoxic T cells (112). Regarding LAG-3 pathway, commercially developed anti-LAG-3-antibody, relatlimab, has been shown to exhibit a limited efficacy as standalone treatment, encouraging its application in combination therapies. Co-treatment with relatlimab and nivolumab is FDA approved, showing lower risk of death and extended progression-free survival. It is important to note that these observations were made in the context of cancer, while their effect on crosstalk between fibroblasts and CD8+ T cells is still in the need of more research. Therefore, without disturbing this interaction, the combination of chemotherapy and immune checkpoint blockade would be inefficient (113).

Recent studies suggest that depletion of ECM components such as beta-ig3 would decrease thickness of the ECM and therefore, allowing T cell movement towards the tumor parenchyma (**Figure 10**). A different approach targeted depletion of fibroblasts using cell surface markers as an effective way to reduce CAF-mediated immunosuppression. Scientists attempted to target FAP and αSMA using DNA vaccines, monoclonal antibodies, genetic depletion, and others. However, depletion of FAP+ stromal cells failed in metastatic colorectal cancer while depletion of αSMA+ fibroblasts resulted in an unexpected more invasive cancer with increase hypoxia and EMT. These observations raise the concerns about the potential challenges associated with characterization of this unique CAF populations.

## Author contributions

AF: Conceptualization, Visualization, Writing – review & editing, Writing – original draft. NA: Conceptualization, Visualization, Writing – review & editing, Funding acquisition, Project administration, Supervision.
